# The West Nile virus genome harbors essential riboregulatory elements with conserved and host-specific functional roles

**DOI:** 10.1073/pnas.2312080121

**Published:** 2024-07-10

**Authors:** Nicholas C. Huston, Lucille H. Tsao, Doug E. Brackney, Anna Marie Pyle

**Affiliations:** ^a^Department of Molecular Biophysics and Biochemistry, Yale University, New Haven, CT 06511; ^b^Department of Chemistry, Yale University, New Haven, CT 06511; ^c^Department of Entomology, Connecticut Agricultural Experimental Station, New Haven, CT 06511; ^d^Department of Molecular, Cellular, and Developmental Biology, Yale University, New Haven, CT 06511; ^e^HHMI, Chevy Chase, MD 20815

**Keywords:** West Nile virus, in vivo SHAPE-MaP, functional RNA structure, genome cyclization, flavivirus

## Abstract

West Nile virus (WNV) is an arthropod-borne, positive-sense RNA virus that poses an increasing global threat due to warming climates and lack of effective therapeutics. Like other enzootic viruses, little is known about how host context affects the structure of the full-length RNA genome. Here, we report a complete secondary structure of the entire WNV genome within infected mammalian and arthropod cell lines. Our analysis affords structural insights into multiple, conserved aspects of flaviviral biology. We show that the WNV genome folds with minimal host dependence, and we prioritize well-folded regions for functional validation using structural homology between hosts as a guide. Using structure-disrupting, antisense locked nucleic acids, we then demonstrate that the WNV genome contains riboregulatory structures with conserved and host-specific functional roles. These results reveal promising RNA drug targets within flaviviral genomes, and they highlight the therapeutic potential of ASO-LNAs as both WNV-specific and pan-flaviviral therapeutic agents.

## Significance

Our work reveals that the West Nile virus (WNV) RNA genome folds with minimal host dependence. We show that a specific genome conformation dominates in cells and propose replication complex formation as a “molecular switch” for transition between life cycle phases. We describe a domain architecture of the 3′ untranslated region (UTR) that provides mechanistic insights into subgenomic flaviviral RNA (sfRNA) production. We identify six riboregulatory elements that play host-specific functional roles. We show that structural homology is an indicator of functional RNA structure, a method extendable to other enzootic viruses. We show that antisense locked nucleic acids (ASO LNAs) can induce potent defects in WNV growth, representing an exciting development in nucleic acid therapeutics.

West Nile virus (WNV) is an arthropod-borne (arbo-) virus with a single-stranded, positive-sense RNA genome. Like other *Flaviviruses,* WNV is maintained in a transmission cycle between a vertebrate reservoir host and invertebrate mosquito vectors. Due to a continued global spread facilitated by a warming climate, *flaviviruses* represent an increasingly serious global health threat (1). As no human vaccine or effective therapeutic exists against WNV, there is a need for research that expands our understanding of WNV biology and facilitates the development of both WNV and pan-*flaviviral* antivirals.

The WNV genome is 11 kb, encodes 10 proteins translated as single polyprotein, and is flanked by 5′ and 3′ untranslated regions (UTRs). WNV UTRs are highly structured, containing RNA motifs that expand the functional repertoire of the virus by mediating crucial steps in the viral life cycle. A conserved structure in the 5′UTR, called the Stem Loop A (SLA) promoter, is required for genome replication by recruiting the viral polymerase (NS5) (2–4). The 3′UTR, one of the best-studied regions of the WNV genome, contains structures that facilitate innate immune evasion and pathogenesis via liberation of a noncoding RNA species, called the subgenomic flaviviral RNA (sfRNA) in a process conserved across flaviviruses (5–7). While structures of sfRNA subdomains have been well studied, it is not known how the structure of the entire 3′UTR affects sfRNA folding and production.

Functional RNA structures in the WNV genome can be dynamic. Complementary regions at the 5′ and 3′ viral termini form long-range duplexes, allowing the genome to alternate between a linear and cyclized conformation (8–10). Genome cyclization is absolutely required for viral replication but is dispensable for viral translation (11). This observation gave rise to a model in which genome cyclization functions as a molecular switch and a subsequent search to identify factors that influence how the genome alternates between linear and cyclized forms. While several groups have separately pointed to both host binding proteins and intrinsic sequence elements as deterministic factors, it has been shown that a balance of linear and cyclized genome conformations is essential for viral fitness (12–15). However, this has never been directly assessed in cells with a full-length WNV genome. Similarly, other functional elements within the WNV open reading frame (ORF) have been identified in silico and validated. However, a comprehensive analysis of functional structures throughout the whole ORF has not been undertaken.

Prior work with both viral and messenger RNAs has highlighted the importance of probing RNAs in their natural cellular context (16–21). As WNV is maintained in an enzootic cycle between vertebrate and invertebrate hosts, a full understanding of its genome structure requires studying how it folds in multiple organismal contexts. Indeed, vertebrate and invertebrate model cell systems have evolutionarily distant host proteomes, varying intracellular salt concentrations, and require culturing temperatures that differ by ~10 °C, all of which are features individually known to have important effects on the folding of functional RNAs (22–24). In fact, careful analysis of dengue and WNV 3′UTRs has identified functional elements that are host specific and thermally responsive, respectively (25–27). To date, no genome-wide study of functional RNA structure in a viral genome has been conducted in multiple hosts.

Here, we report the complete secondary structure of the WNV genome in arthropod and mammalian cell lines using Selective 2’-Hydroxyl Acylation analyzed by Primer Extension and Mutational Profiling (SHAPE-MaP) (28). The SHAPE-MaP data are of exceptional quality, and the resulting genomic secondary structure model perfectly recapitulates the well-characterized SLA motif in the 5′UTR, which benchmarks the study. The data enable us to examine genome cyclization in vivo and elucidate the natural conformational dynamics of the WNV genome in infected cells. We identify a tripartite domain architecture at the 3′ viral terminus, highlighting the importance of studying viral RNA structures in their native genomic context. We describe a genome that folds with minimal host dependence, and, using patterns of RNA structural homology between hosts, we prioritize specific well-folded RNA structures for downstream functional validation. Using structure disrupting, antisense locked nucleic acids (ASO LNAs), we demonstrate that a subset of these well-folded regions play both conserved and host-specific functional roles, and we then test a subset of these genetically. Our work deepens the understanding of WNV biology and identifies conserved aspects of the viral life cycle that are readily targetable by antisense nucleic acids. As such, it represents an important step forward in our fight against an expanding global health threat.

## Materials and Methods

### Cell Culture.

Vero cells (American Type Culture Collection (ATCC), CCL-81) were cultured in Dulbecco’s Modified Eagle Medium (DMEM) supplemented with 10% heat-inactivated fetal bovine serum (HI FBS) supplemented with nonessential amino acids (NEAA), L-glutamine, and sodium bicarbonate and incubated at 37 °C/5% CO_2_. C6/36 cells (ATCC, CRL-1660) were cultured in DMEM supplemented with 10% HI FBS DMEM, NEAA, L-glutamine, and sodium bicarbonate and incubated at 28 °C/5% CO_2_. Unless otherwise stated, cell lines were cultured as described above. All experiments using live WNV were performed in the BSL3 facility at the Connecticut Agriculture Experimental Station.

### Construct Design and Preparation.

Disruption of the cyclized (CYC) duplex by inverting five consecutive nucleotides in the 3**′**CYC arm results in a replication-incompetent virus (8). To generate this mutant, we introduced mutations into the full-length infectious cDNA clone using site-directed mutagenesis (SDM). SDM primers can be found in *SI Appendix*, Table S1. Successful mutagenesis was verified by sequencing at the Yale Keck Facility.

To generate Region 11 and Region 12 mutant genomes, synonymous mutations were designed in the corresponding regions so as to result in maximal secondary structure disruption without altering coding potential. Inserts that contained the engineered mutations were obtained from ThermoFisher Scientific. Using sticky end ligation, mutant inserts were ligated back into the full-length, wild-type plasmid. Successful mutagenesis was verified by whole plasmid sequencing at Quintara Biosciences.

### In Vivo SHAPE-MaP, RT, and Library Preparation.

A plasmid containing the full-length infectious cDNA clone of the WNV NY99 (AF404756) was used to generate full-length genomic RNA. Briefly, the plasmid was linearized using Xba1 (New England Biolabs (NEB), Cat. No. R015S), followed by ethanol precipitation and resuspension in Tris-EDTA (TE) pH 7.5. The linearized plasmid serves as a template in a run-off, in vitro transcription using T7 RNA polymerase variant P266L (29) with reaction conditions previously described (30). Following transcription, plasmid template was digested with RQ1 DNase (Promega, Cat. No. M6101), and RNA was purified using the RNeasy kit (Qiagen, Cat. No. 74104) according to the manufacturer’s protocols. A type-1 cap, including an inverted 7-methylguanosine and a 2’-OMe on the first nucleotide, was added to the RNA using the Vaccinia Capping Enzyme (NEB, Cat. No. M2080S) and 2’-OMe transferase (NEB, Cat. No. M0366S) according to the manufacturer’s protocols. Capped RNA was purified using an RNeasy column and eluted in 1× ME buffer (8 mM 3-(N-morpholino)propanesulfonic acid (MOPS) and 0.1 mM ethylenediaminetetraacetic acid (EDTA), pH 6.5). Capped RNA was visualized on a denaturing agarose gel to ensure production of full-length products.

Prior to transfection, Vero or C6/63 cells were seeded into eight 10 cm tissue culture treated plates and grown to ~90% confluency. Transfection of in vitro transcribed, capped viral RNA was performed using the Mirus *TransIT®-mRNA Transfection Kit* (Cat. No. MIR2225). Specifically, 6 µg of viral RNA was transfected per 10 cm plate according to the manufacturer’s protocol. Four hours posttransfection (hpt), cells were gently washed once with cold 1× Phosphate-buffered saline (PBS), and complete media were replaced.

Four days postinfection (dpi), media was aspirated from tissue culture plates, and cells were washed once with cold 1× PBS. The contents of 4 × 10 cm plates were collected in 2 mL of 1× PBS, pelleted at 1,000 g for 5 min at 4 °C, and resuspended in 2 mL 1× PBS. Subsequently, 200 µL of 2 M 2-methylnicotinic acid imidazolide (NAI) or an equivalent volume of DMSO was added to the suspension. Samples were pipetted vigorously to ensure sufficient mixing and incubated in the dark for 10 min. Following incubation, 6 mL of Trizol reagent was added. Samples were incubated for 10 min to ensure complete viral inactivation. RNA was extracted with addition of chloroform:isoamyl alcohol (24:1) and subsequently precipitated. RNA pellets were resuspended in 1× ME buffer, purified using the Qiagen RNeasy kit according to the manufacturer’s protocol, and stored at −80 °C until needed.

To prepare sequencing libraries, we relied on a tiled-amplicon approach. Specifically, we designed 19 amplicons that were 700 nucleotide (nts) in length and which were tiled across the WNV genome to achieve full sequencing coverage. Adjacent amplicons overlapped by 100 nt, with additional overlap at the 5′ and 3′ viral termini to ensure sufficient sequencing coverage. A list of all gene-specific RT and PCR primers used is available in *SI Appendix*, Table S1.

RT reactions were prepared using 1.5 μg of total cellular RNA, SuperScript II (SSII) (Invitrogen, Cat. No. 18064014), SSII-MaP reaction buffer (50 mM 1 M Tris-HCl pH 8.0, 75 mM KCl, 10 mM Dithiothreitol (DTT), 6 mM MnCl_2_, and 0.5 mM deoxynucleotide triphosphate (dNTP)), and 1 μM gene-specific RT primer. RT reactions were incubated at 42 °C for 3 h. Following RT, viral RNA was degraded enzymatically at 37 °C using an equal mix of RNaseA (NEB, Cat. No. T3018L), RNaseT1 (NEB, Cat. No. EN0541), and RNaseH (NEB, Cat. No. M0297S). Single-stranded cDNA was purified using AmpureXP beads (Agencourt, Cat. No. A63881) with a bead to sample ratio of 1.8:1.

Tiled amplicons were generated using 5 μL of cDNA, gene-specific forward and reverse primers, and NEBNext Ultra II Q5 Mastermix (Cat. No. M0544L). Touchdown cycling PCR conditions were used to enhance PCR specificity (68 to 58 °C annealing temperature gradient) (31). PCR products were purified with Monarch PCR & DNA Cleanup Kits (NEB, Cat. No. T1030S) with a binding buffer:sample ratio of 2:1. Even and odd tiled amplicons were subsequently pooled for downstream library preparation.

### In Vitro SHAPE-MaP, RT, and Library Preparation.

In vitro transcription of genomic WNV RNA was performed as described above. After transcription, plasmid template was digested using RQ1 DNase, followed by addition of 30 mg/mL Proteinase K (ThermoFisher, Cat. No. 17916) to inactivate all enzymes. To this reaction was added 25 mM final EDTA at pH 8.0 to chelate Mg^2+^. Samples were applied to a 100 kDa Amicon Ultra filtration column (Amicon, Cat. No. UFC510096) and spun to half volume. Filtration buffer (50 mM 2-(4-(2-hydroxyethyl)-1-piperazinyl)-ethanesulfonic acid (K-HEPES) pH 7.5, 150 mM KCl, and 100 μM EDTA pH 8.0) was added to the sample and spun to half volume. This step was repeated a total of eight times to ensure removal of unincorporated NTPs and products of enzymatic digestion.

Subsequently, RNA was subjected to size-exclusion chromatography, performed at room temperature, using a self-packed Sephacryl-1000 column with a 24 mL bed volume pre-equilibrated with filtration buffer. RNA from the peak fraction was diluted to 100 ng/µL and folded in the presence of 10 mM Mg^2+^ at 37 °C for 30 min. Following folding, RNA was modified with a final concentration of 10 mM 1M7 for 3 min at 37 °C (synthesized in-house). Probing reactions were quenched and precipitated with EtOH and resuspended in 1× ME.

RT reactions were conducted using 1 μg of in vitro purified RNA, SSII, SSII-MaP reaction buffer (50 mM 1 M Tris-HCl pH 8.0, 75 mM KCl, 10 mM DTT, 6 mM MnCl_2_, and 0.5 mM dNTP), and random nonamers (NEB, Cat. No. S1254S). RT reactions were incubated at 42 °C for 3 h. Second strand synthesis was performed using the NEBNext Ultra II Non-Directional Second Strand synthesis module (NEB) according to the manufacturer’s protocol. Double-stranded complementary DNA (cDNA) was purified using Monarch DNA cleanup kits and a 5:1 binding buffer:sample ratio.

## Library Quantification, Sequencing, and Data analysis.

Following generation of double-stranded cDNA in vitro libraries or dsDNA in vivo odd and even amplicon pools, samples were diluted to 0.2 ng/μL. Libraries were fragmented and tagged with Illumina sequencing adaptors using the NexteraXT DNA library preparation kit (Illumina, Cat. No. FC-131-1024) according to the manufacturer’s protocols, but at 1/5th the recommended volume.

Library concentration and average size were quantified with a Qubit dsDNA HS Assay Kit (ThermoFisher, Cat. No. Q32851) and a BioAnalyzer High Sensitivity DNA Analysis kit (Agilent, Cat. No. 5067-4636), respectively. Library concentration and average size were used to dilute libraries to 4 nM which were subsequently denatured. Final library dilutions were prepared according to the manufacturer’s protocols, and libraries were sequenced on the NextSeq 500/550 platform using a paired-end, dual-indexed 150-cycle sequencing kit.

All sequencing data were analyzed using ShapeMapper 2.0 (32), aligning reads to the WNV genome (AF404756) using the default alignment parameters. Sequencing data analysis did not include primer masking options because libraries were constructed using overlapping amplicons and Nextera Tagmentation. Default quality control benchmarks implemented in ShapeMapper2.0 were used to ensure that data were of high quality and are available in *SI Appendix*, Table S2. Mutation rates of 1M7- or NAI-modified samples were compared to unmodified samples and tested for significance using the equal variance *t* test.

### Structure Prediction.

ShapeKnots was used to examine the WNV genome for pseudoknots (PK) (33). Specifically, in silico predictions were performed across the entire genome in 500 nt windows separated by a 100 nt slide. For each window, the coordinates of PKs that appeared in the five most stable pseudoknotted structures were extracted. PKs were considered plausible if a given PK appeared in the majority of extracted pseudoknotted structures in all of the windows that covered the predicted region. In addition to identifying five novel PKs, these filtering criteria successfully capture two previously reported PKs, including a programmed ribosomal-frameshifting PK contained in NS4B (34) and an exoribonuclease-resistant PK found in the 3′UTR (7, 35).

Superfold (36) was used to generate a unique consensus structure prediction for each replicate of in vitro and in vivo SHAPE data collected. Normalized reactivities were included as experimental constraints using default slope and intercept values and a maximum pairing distance of 500 nt. PKs flagged with ShapeKnots were only included as hard constraints if the majority of pseudoknotted nucleotides had low reactivity (<0.4) in the corresponding datasets. Four additional PKs, for which an abundance of functional data exist, were included as hard constraints after evaluation using the same criteria (*SI Appendix*, Fig. S2) (5, 7, 26, 35, 37). A list of PKs included in all SuperFold predictions is detailed in *SI Appendix*, Table S3. Structure output from the SuperFold prediction pipeline was visualized using StructureEditor, a tool in the RNAstructure software suite (38). Full-length structures (.ct file) and SHAPE reactivities from Vero and C6/36 models are available at the PyleLab GitHub repository.

### Identification of Well-Folded Regions.

We relied on two SHAPE reactivity and Shannon entropy data signatures to identify regions that are highly structured and stably folded, respectively. SHAPE reactivity is calculated using the ShapeMapper analysis tool described above. Shannon entropy is derived from base-pairing probabilities calculated using the SuperFold partition function (36). Each replicate SHAPE dataset collected per cell type was used to generate separate SuperFold predictions.

For a given cell type, local median SHAPE reactivity and Shannon entropy were calculated in 55 nt sliding windows. The global median SHAPE reactivity and Shannon entropy were subtracted to facilitate subsequent analysis steps. Regions were considered “well folded” if they met two criteria; 1) local SHAPE and Shannon entropy signals were below the global median for stretches ≥40 nts and 2) these regions appeared in both replicate datasets. Regions were included even if the local SHAPE reactivity or Shannon entropy rose above the global median as long as these stretches were <40 nts. Replicate consensus structure predictions were compared for regions that meet the above criteria to ensure agreement between structure models. Well-folded regions identified in each cell type are reported in Table 1.

**Table 1.** Database of well-folded regions identified in WNV

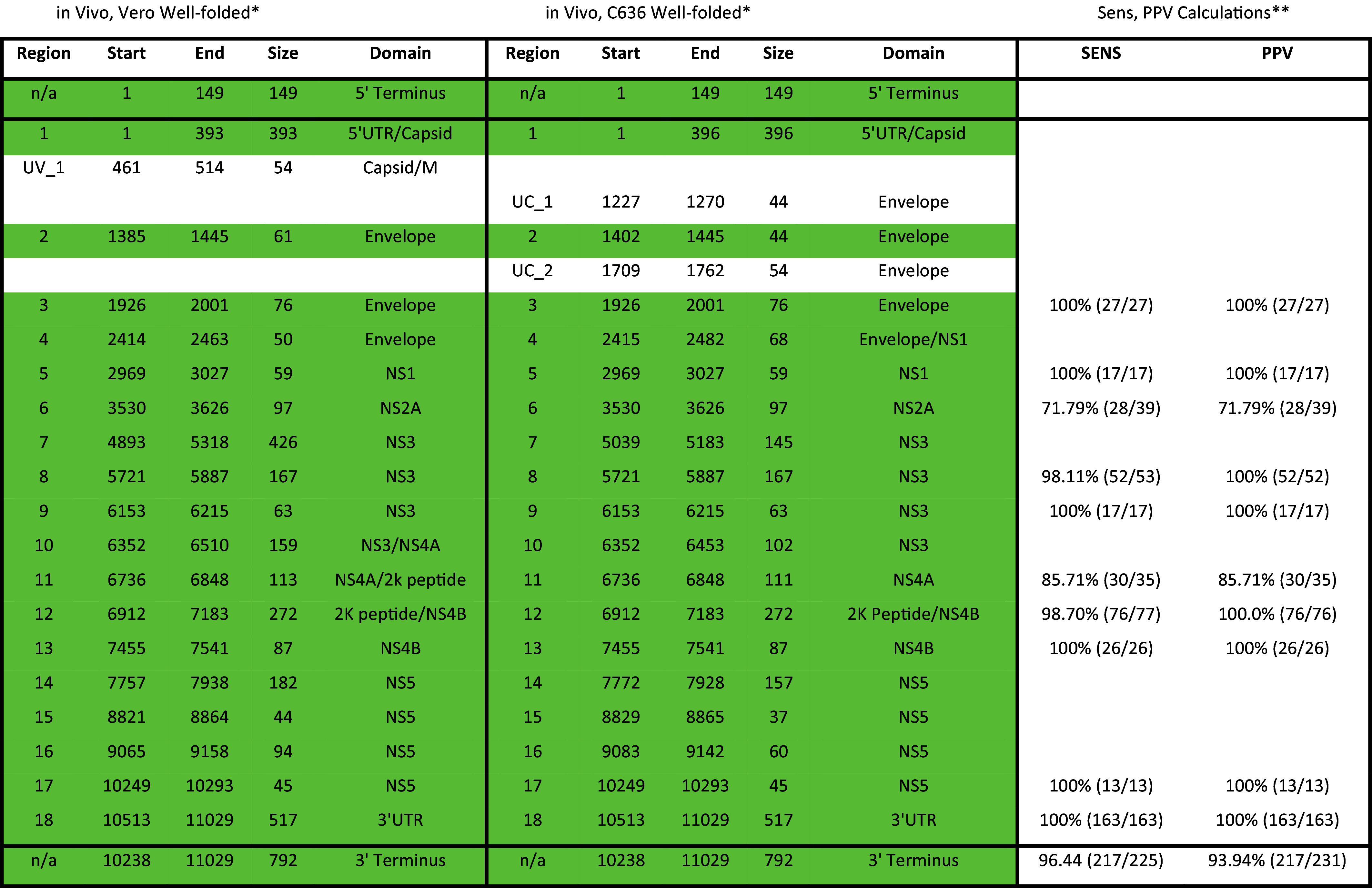	

^*^Shaded dark green = identical nucleotide boundaries; shaded light green = overlapping nucleotide boundaries; not shaded = cell type specific.

^**^For Sensitivity (SENS) and Positive Predictive Value (PPV) calculations, Vero structure = “Predicted”, C6/36 structure = “Accepted”.

### Multiple Sequence Alignment (MSA).

To analyze evolutionary support for consensus structure predictions generated in either Vero or C6/36 cell types, we compiled a codon-based MSA for genomes of mosquito-borne *flaviviruses*. All sequences were chosen based on a phylogenetic study of *flaviviruses* (39). Sequences included in the MSA were downloaded from NCBI and are detailed in *SI Appendix*, Table S4.

The open-reading frames of each virus were extracted from the full-length sequences based on the GenBank annotations. Codon alignments were generated using MACSE v2.0.3 (40) and default parameters (-prog alignSequences).

### Synonymous Mutation Rate Analysis.

Codon alignments generated with MACSE were visualized with Jalview v2.11.0 (41). Sequences that corresponded to gaps in the parental WNV NY99 sequence were deleted. Synonymous mutation rates for each codon in the WNV genome were estimated using the phylogenetic-based parametric maximum likelihood (FUBAR) method (42). Using a representative consensus structure prediction derived from either Vero or C6/36 cells, each codon was categorized as single or double stranded as determined by the strandedness of the nucleotide in the third position. Statistically significant differences between synonymous mutation rates separated into single- and double-stranded bins were determined using the two-tailed, equal variance *t* test.

### ASO LNA Design and Transfection.

ASO LNAs were designed to anneal to target sequences within the WNV genome. All ASO LNAs were designed with three consecutive LNA bases at the 5′ and 3′ ends, with internal stretches of unlocked bases limited to three consecutive nucleotides (*SI Appendix*, Table S5). The ASO LNAs were designed with similar thermodynamic properties, such as length, %GC content, %LNA content, and LNA:RNA duplex T_m_. All ASO LNA oligos were synthesized on a MerMade 12 RNA-DNA synthesizer (BioAutomation) using DNA and LNA phosphoramidites (TxBio) and Glen UnySupport 1000 (Glen Research). The synthesis cycle was modified to increase oxidation step to 3 min as recommended by the manufacturer. Removal of oligonucleotides from the polymer support and base deprotection were carried out in 28 to 30% aqueous ammonium hydroxide: 40% aqueous methylamine (1:1) at 65 °C for 2 h. Then, the resulting oligo solutions were dried using Speedvac, redissolved in 750 µL of sterile deionized water, and desalted using GlenPak 1.0 columns (Glen Research).

Prior to ASO LNA transfection, Vero and C6/36 cells were plated and grown to ~90% confluency in 12-well tissue culture plates. ASO LNAs were cotransfected in quadruplicate at a final concentration of 400 nM/well along with 0.5 μg of in vitro transcribed, Type-1 capped wild-type WNV genomic RNA (WNVic) or Region 11 and Region 12 mutant genomes alone. Four hpt, cells were washed with cold 1× PBS, and complete media was replaced.

Supernatant samples were collected on 3 dpi from Vero cells or 6 dpi from C6/36 cells. Samples were spun down at 1,000 g for 5 min at 4 °C to remove any cellular debris. To remove any RNAs not encapsulated in virions, supernatant samples were subjected to a 30 min RNase A degradation at 37 °C. RNase A was deactivated with addition of 20 mg/mL Proteinase K and incubation at 37 °C for 1 h. Viral RNA was extracted from lysed, inactivated virions using the Mag-Bind Viral DNA/RNA 96 Kit (Omega Bio-Tek, M6246) and a Kingfisher Flex liquid-handling robot and frozen at −80 °C prior to use.

### Quantification of Viral Genomes.

To monitor viral growth, we relied on a quantitative RT-PCR assay adapted from (43). We first prepared a 2.4 kb RNA standard from a portion of the WNV genome that includes the E gene by performing T7 transcription from a PCR template generated from a plasmid containing the full-length infectious cDNA clone (Genome Coords: 1031-3431). RNA standards were purified using an RNeasy column per the manufacturer’s instructions, quantified, and aliquoted in serial 10-fold dilutions.

Viral RNA copy numbers in supernatant samples were determined using a 6-carboxy-fluorescein (FAM)-labeled TaqMan probe targeted against the E gene, primers WNV1160F and WNV1229R, and the Luna Universal Probe One-Step RT-qPCR kit (NEB, Cat. No. E3006L). Using a linear regression derived from the standard curve, we calculated viral RNA genome copy number per microliter of supernatant, and statistical outliers were removed using the ROUT outlier test available in GraphPad Prism. Primer and probe sequences are available in *SI Appendix*, Table S1.

## Results

### In Vivo Pipeline Yields High-Quality SHAPE-MaP Data from Two Cell Types.

**Fig. 1.**
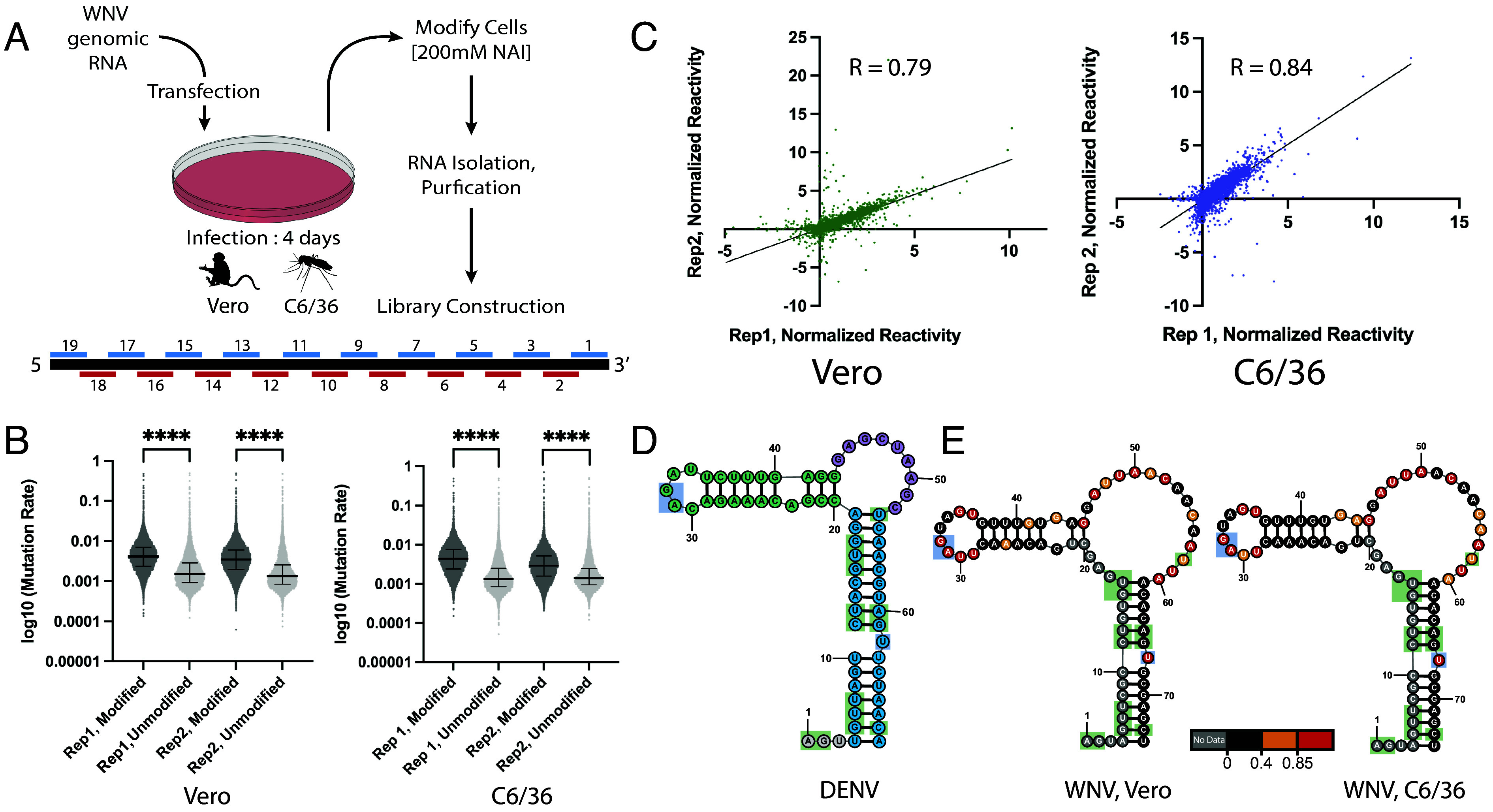
Tiled amplicon SHAPE-MaP workflow yields high-quality in vivo reactivity data from multiple cell types, and de novo structure prediction recapitulates a conserved functional motif (*A*) Workflow of in vivo SHAPE-MaP probing using in vitro transcribed WNV RNA to initiate infection in Vero and C6/36 cells. RNA is modified with NAI 4 dpi, and a tiled-amplicon approach is used to achieve full genome coverage. (*B*) Comparison of mutation rates of NAI-modified or unmodified samples for two independent biological replicates in both Vero and C6/36 cells. Lines indicate the mean, and whiskers indicate the SD. ****P < 0.0001 by equal variance unpaired Student’s *t* test. (*C*) Correlation plot of normalized SHAPE reactivities from two biological replicates collected in either Vero or C6/36 cells. Lines represent linear regressions fit to the data. Pearson’s correlation for each dataset is shown. (*D*) Secondary structure of dengue virus SLA promoter, adapted from ref. 2. Bottom stem = blue; top stem = teal; side loop = purple. (*E*) Secondary structure prediction of WNV SLA extracted from full-length prediction in Vero (*Left*) or C6/36 (*Right*) cells, colored by SHAPE reactivity. Green-shaded nucleotides = conserved; blue-shaded nucleotides = conserved, functional.

WNVic was in vitro transcribed from an infectious clone of the NY99 strain (44), capped with a Type 1 cap, and transfected into either Vero or C6/36 cells. At 4 dpi, cells were collected and modified with NAI, an electrophilic reagent that reacts preferentially with 2’OH moieties in flexible regions of the RNA backbone, or DMSO as a treatment control (Fig. 1*A*) (45). Following extraction and purification of RNA, sequencing libraries were generated using nineteen 700 nt amplicons tiled across the WNV genome, with 100 nt overlap between adjacent amplicons (Fig. 1*A*). RT was performed with gene-specific RT primers and manganese, a non-natural cofactor that allows for the encoding of RNA-adducts as cDNA mutations (28). Two independent biological replicates were prepared for each cell type.

Resulting SHAPE-MaP data were subjected to stringent quality control metrics to ensure that data were of sufficient quality for de novo structure prediction (46). Median effective read depth was >55,000× in both Vero replicates and >35,000× in both C6/36 replicates, far exceeding the read depth threshold required for high confidence reactivity calling (36). As a result, we collected effective reactivity data for 99.8% and 99.7% of the WNV genome in infected Vero or C6/36 cells, respectively. Mutation rates of modified samples were significantly elevated relative to unmodified samples in all four replicates analyzed (Fig. 1*B*; *P*-value < 0.0001), confirming that WNV RNA was successfully modified with NAI in both in vivo contexts and that these adducts were encoded as cDNA mutations. Finally, strong correlation was observed across the entire WNV genome in both cell types (Fig. 1*C*; Vero = 0.79; C6/36 = 0.84), verifying that these biophysical measures of RNA backbone flexibility are highly reproducible and that SHAPE-MaP data are of sufficient quality for genome-wide structure prediction.

We relied on the SuperFold pipeline to generate genome-wide structure predictions using the reactivities generated in vivo as experimental constraints (36). The secondary structure model of the DENV SLA derived from its crystal structure includes a bottom stem (blue), a top stem (teal), and a single-stranded side loop (purple), that are not correctly resolved when performing unconstrained secondary structure predictions (2) (Fig. 1*D*). Both in vivo secondary structure models of the WNV SLA, generated either in Vero or C6/36 cells, recapitulate this overall architecture, along with the positions of absolutely conserved nucleotides with both known and unknown function (Fig. 1*E*, shaded blue or green, respectively). Together, these findings suggest that our data and structure predictions are of high quality and can confidently be used to identify novel riboregulatory elements.

### SHAPE Data Reveal that Linear Genome Conformation Dominates In Vivo while Cyclized Conformation Dominates In Vitro.

**Fig. 2.**
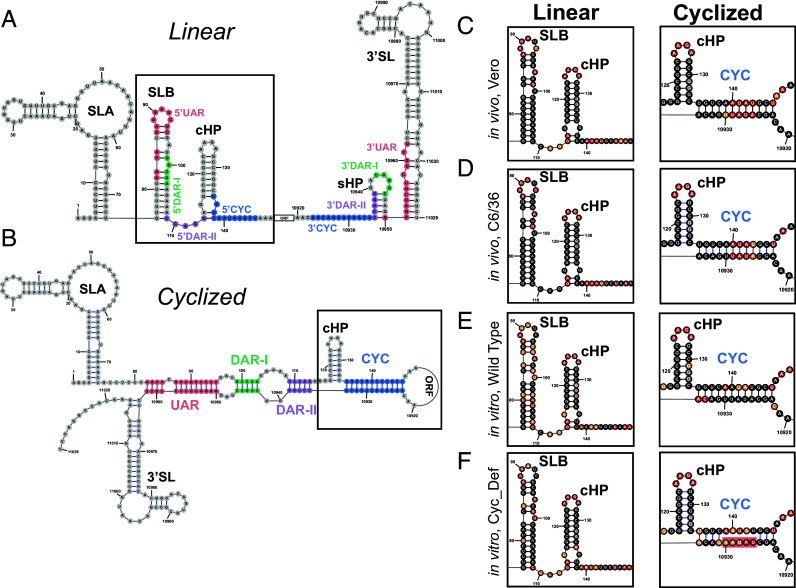
The WNV genome favors the linear genome conformation over the cyclized genome conformation in infected mammalian and arthropod cell lines. (*A*) Schematic of secondary structures at the 5′ and 3′ viral termini that comprise the linear genome conformation. Sequences that mediate genome cyclization are colored and labeled. SLA, SLB, Capsid Hairpin (cHP), ORF, Short Hairpin (sHP), 3′SL. (*B*) Schematic of secondary structures and long-range duplexes that comprise the cyclized genome conformation, labeled as in *A*. (*C*–*F*) Normalized SHAPE reactivities mapped to mutually exclusive structural elements of the linear (*Left*) or cyclized (*Right*) genome conformation, labeled as in *A* or *B*, respectively. Cyclization defection (Cyc_Def); Mutated nucleotides are shaded red.

The WNV genome alternates between two conformations, linear or cyclized, in a process called genome cyclization (47). This process is mediated by four pairs of complementary sequences that form noncontiguous, long-range duplexes between the extreme 5′ and 3′ viral termini (Fig. 2 *A* and *B*). These critical sequences have varying degrees of structure depending on the genome conformation. While the secondary structures that comprise the linear conformation can be extracted from structure predictions prepared above, the cyclized genome conformation cannot be predicted due to distance constraints imposed during structure prediction (48). We therefore evaluated the mapping quality of our in vivo SHAPE reactivity to determine which of the mutually exclusive conformations, linear or cyclized, dominates in vivo.

Mapping the in vivo SHAPE-MaP data from both cell types to the predicted SLB structures, which only fold in the linear genome conformation, revealed that highly reactive nucleotides are almost entirely restricted to single-stranded regions, while lowly reactive nucleotides are restricted to double-stranded regions (Fig. 2 *C* and *D*, Linear). Conversely, when mapped to the cyclized conformation, highly reactive nucleotides are notably found within the predicted CYC duplex (Fig. 2 *C* and *D*, Cyclized). Such findings are consistent with the WNV genome favoring a linear conformation in vivo.

Because multiple host proteins have been implicated in genome cyclization, we performed in vitro probing experiments on natively purified, full-length genomic RNA to better understand the cyclization dynamics of the genomic RNA in a protein-free system (4, 12, 49). Reactivities derived from the wild-type WNV construct show that the cyclized genome conformation dominates in vitro (Fig. 2*E*). This is evidenced by strong disagreement between our in vitro reactivity data and a folded SLB, including highly reactive nucleotides at the base of SLB that become single-stranded upon genome cyclization (Fig. 2*E*, Linear). Even more, low reactivity values support the formation of the long-range CYC duplex, including nucleotides that were shown to have high reactivity in vivo (Fig. 2*E*, Cyclized).

Importantly, the use of an in vitro system allows us to probe a cyclization defective mutant (Cyc_Def) that cannot grow in cells, and therefore cannot be structurally probed. The Cyc_Def mutant is generated by inverting five nucleotides in the 3′CYC duplex, thus disrupting formation of the CYC duplex (Fig. 2*F*; mutated nucleotides shaded red). This mutation renders the virus replication-incompetent and unable to revert. Our in vitro probing data of the Cyc_Def mutant reveals reactivity mapping to SLB that supports the presence of the linear conformation, while reactivity mapping to the CYC duplex shows highly reactive nucleotides present in both the 5′ and 3′CYC duplex arms (Fig. 2*F*). These data further confirm that the linear genome conformation dominates in vivo, as the in vivo reactivity of the actively replicating WNV perfectly recapitulates the reactivity of the Cyc_Def construct in vitro. These data suggest that in the absence of any host factors, the WNV genome naturally favors the cyclized genome conformation. In a cellular context, host factors present under the growth conditions we examined may disrupt this natural equilibrium, causing the genome to favor its linear form.

### Genome-Wide Structure Prediction of the WNV Genome Reveals a Tripartite Domain Architecture of the 3′ Viral Terminus.

**Fig. 3.**
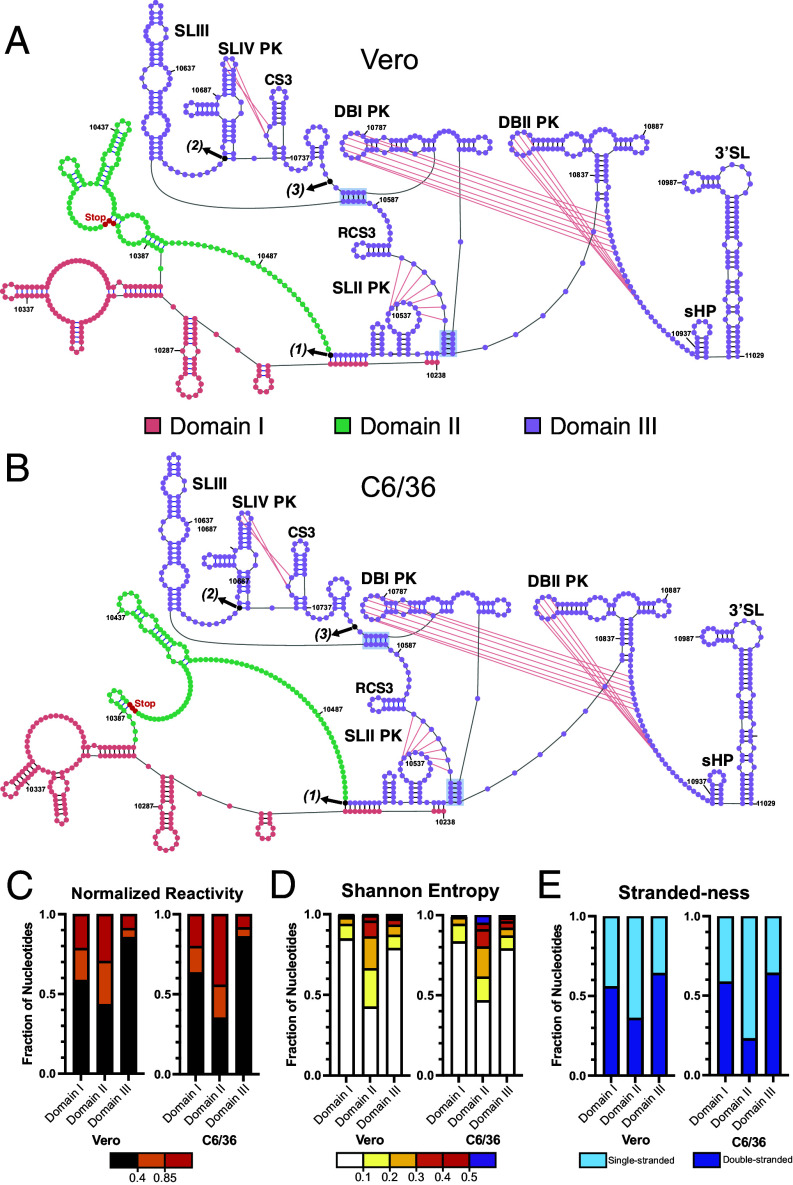
The 3′ viral terminus of WNV is composed of three distinct RNA structural domains. Structure of the 3′ viral terminus determined in infected (*A*) Vero or (*B*) C6/36 cells, color-coded by domain. The stop codon is colored in red; the starting positions of sfRNA1, sfRNA2, and sfRNA3 are indicated with black arrows that are labeled with their corresponding sfRNA numbers in parentheses; Pseudoknotted bases indicated by pink lines; previously identified structural motifs are labeled. (*C*) Nucleotides in each domain binned by normalized reactivity, with bin size expressed as a fraction of total nucleotides in that domain. (*D*) Shannon entropy signatures of each domain, plotted as in *C*. (*E*) Strandedness of each domain, plotted as in *C*.

Our in vivo data support the presence of four previously identified PKs known to play important roles in sfRNA production (5, 7, 26, 35). Indeed, the majority of all pseudoknotted nucleotides are lowly reactive in both replicate datasets in both cell types (*SI Appendix*, Fig. S1 *A* and *B*). These PKs were forced in all genome-wide structure predictions that were performed. The structure of the 3′ viral terminus, extracted from these genome-wide predictions, reveals a tripartite domain architecture with striking structural homology between cell types (Fig. 3 *A* and *B*). Each of the three structural domains is identified on the basis of a unique and reproducible combination of SHAPE reactivity, Shannon entropy, and strandedness.

Domain I (Fig. 3 *A* and *B*, pink) is situated upstream of the stop codon in the coding region of NS5. The majority of the nucleotides contained in Domain I exhibit low reactivity, low Shannon entropy, and are double-stranded; ~60% of nucleotides have reactivities <0.4 (Fig. 3*C*; Vero = 58.6%, C6/36 = 63.4%), ~85% of nucleotides have Shannon entropy <0.1 (Fig. 3*D*; Vero = 84.8%, C6/36 = 83.4%), and ~55% of nucleotides are double-stranded (Fig. 3*E*; Vero = 55.9%, C6/36 = 58.6%). In fact, a subsection of this domain qualifies as well-folded, as defined in the methods section (Table 1; Vero—Region18, C6/36—Region19). Most interestingly, the 5′ end of Domain I is engaged in a long-distance interaction with the 5′ end of Domain III. This reveals that the 3′UTR does not fold independently of the viral ORF, confirming a similar observation made regarding the dengue virus 3′ viral terminus (50).

Domain II includes both coding and noncoding nucleic acid sequence and therefore contains the stop codon of the viral ORF. Further, it is unique among the three domains in that it is the most flexible and least folded (Fig. 3 *A* and *B*, green). The majority of the nucleotides contained in Domain II exhibit high reactivity, high Shannon entropy, and are single-stranded; <45% of nucleotides have reactivities <0.4 (Fig. 3*C*; Vero = 43.4%, C6/36 = 35.2%), ~45% of nucleotides have Shannon entropy <0.1 (Fig. 3*D*; Vero = 42.6%, C6/36 = 46.7%), and ~35% of nucleotides are double-stranded (Fig. 3*E*; Vero = 36.1%, C6/36 = 23%). This region was previously reported to include Stem Loop I (SLI), although it is absent from all structural models generated (7). Instead, this region includes a long, highly reactive single-stranded region that sits immediately upstream of Domain III (*SI Appendix*, Fig. S2 *A* and *B*).

Domain III corresponds to the sfRNA and exhibits data signatures that suggest it is the most structured domain at the 3′ viral terminus (Fig. 3 *A* and *B*, purple). The majority of the nucleotides contained in Domain III exhibit low reactivity, low Shannon entropy, and are double-stranded; ~85% of nucleotides have reactivities <0.4 (Fig. 3*C*; Vero = 85.5%, C6/36 = 86.1%), ~80% of nucleotides have Shannon entropy <0.1 (Fig. 3*D*; Vero = 78.9%, C6/36 = 79%), and 64.2% of nucleotides are double-stranded (Fig. 3*E*; Vero = 64.2%, C6/36 = 64.2%). (Fig. 3 *A* and *B*, purple). Strikingly, the structure of Domain III is identical in both cell types (Table 1, Vero – Region 19, C6/36 – Region 20; SENS = 100%, PPV = 100%).

The model we observe deviates slightly from existing models of the WNV sfRNA. As mentioned above, our data indicate that a long-range duplex forms between Domains I and III. Formation of this duplex liberates nucleotides that would otherwise form the base of the SLII, allowing them to engage in previously unreported, longer-range interactions (Fig. 3 *A* and *B*, shaded blue). As a direct consequence, the duplex reported to form the base of Dumbbell I (DBI) also does not fold, instead base-pairing with sequence ~100 nt upstream (Fig. 3 *A* and *B*, shaded gray). Our models otherwise recapitulate all the structural elements previously reported for the WNV 3′UTR, including RCS3, SLIII, SLIV, CS3, DBII, short hairpin, and the 3′SL (Fig. 3 *A* and *B*, labeled). Overall, our model reveals that Domain III is extensively base paired and highly compact and suggests a context-specific fold for both the 3′UTR and the sfRNA.

### The WNV Genome Folds into Networks of Well-Folded Regions with Little Apparent Host Dependency.

**Fig. 4.**
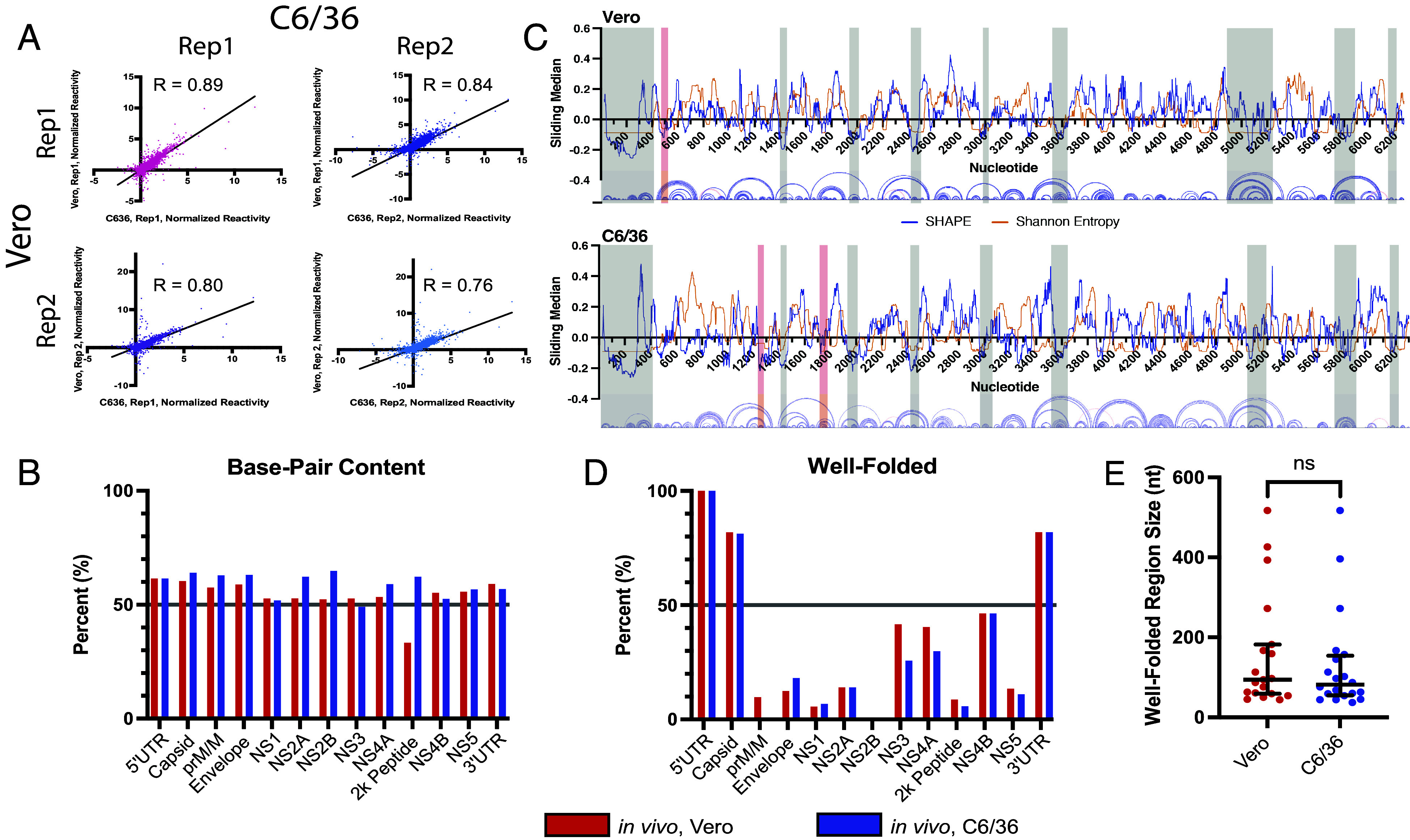
The WNV genome folds into networks of well-folded regions with little apparent host dependency (*A*) Comparison of normalized SHAPE reactivities made between biological replicates collected in either Vero or C6/36 cells. Lines represent linear regressions fit to the data. Pearson’s correlation for each dataset is shown. (*B*) Base-pairing content in the UTRs and individual protein domains determined in Vero (red) or C6/36 (blue) cell types. (*C*) Analysis of SHAPE reactivities and Shannon entropy reveals the presence of highly structured, well-determined domains in WNV. Nucleotide coordinates are indicated on the *x*-axis—only the first half of the WNV genome is shown. Local median SHAPE reactivity and Shannon entropy are indicated by blue and orange lines, respectively. Well-folded regions that appear in both or only a single cell type are shaded with gray or red boxes, respectively. Arc plots for predicted base-pairing interactions in the structural model are shown below the *x*-axis. (*D*) Well-folded RNA content in the UTRs and individual protein domains determined in Vero (red) or C6/36 (blue) cell types. (*E*) The size of well-folded regions determined in Vero (red) or C6/36 (blue) cell types.

Similar to the 5′ and 3′ UTR, we examined whether structural homology extended into the WNV ORF. Analysis of global Vero and C6/36 reactivities revealed a high degree of correlation between the cell types (Fig. 4*A*, 0.76 ≤ R ≤ 0.89; Fig. 1*C*, 0.79 ≤ R ≤ 0.84). Further, we did not observe large differences in the base-pair content (BPC) of the WNV genome, with an average double-stranded content of 54.3% (±7.1%) or 59.0% (±5.2%) across protein domains in Vero and C6/36 cells, respectively (Fig. 4*B*). This suggests that the per-nucleotide backbone flexibility across the entire WNV genome is largely cell-type independent.

To identify riboregulatory structures, we identified regions with local SHAPE reactivity and Shannon entropy below the global median for ≥40 nt that appear in both replicate datasets from either Vero or C6/36 cells. Any region that meets these “lowSS” criteria is hereafter referred to as well folded (20, 21, 28, 50). When comparing the well-folded RNA content between cell types, we observe minimal host dependence. In total, we identified 19 well-folded regions across the WNV genome in Vero cells, and 20 well-folded regions in C6/36 cells (Fig. 4*C* and *SI Appendix*, Fig. S3, shaded boxes). Eighteen appear in both cell types, ten of which have identical nucleotide boundaries, with the remaining eight overlapping (Table 1, shaded dark or light green, respectively). Of all the well-folded regions identified, only 3 three well-folded regions appear in a single cell type (Fig. 4*C*, shaded red boxes). These three regions are small (<55 nt) and cluster exclusively at the 5′ end of the genome. It follows, therefore, that we do not observe large differences in the percent of each protein domain that are well folded (Fig. 4*D*). Furthermore, we did not find significant differences in the size of well-folded domains identified in each cell type (Fig. 4*E*). Taken together, these data demonstrate that WNV folds largely independent of host context and suggest that much like proteins, nucleic acid structure is hard-coded in primary sequence.

### Structural Homology of Well-Folded Regions Serves as a Sorting Criterion for Functional Validation.

**Fig. 5.**
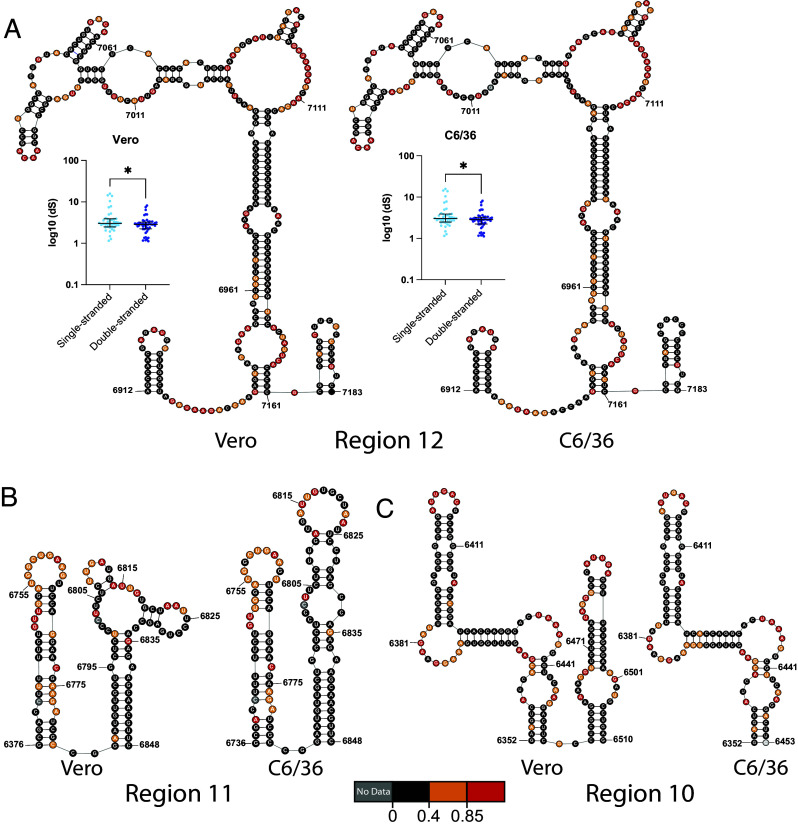
Patterns of structural homology of well-folded regions between cell types allow for prioritization of putative riboregulatory elements (*A*) RNA secondary structure of well-folded Region 12, which has identical nucleotide boundaries and near identical connectivity in Vero (*Left*) and C6/36 (*Right*) cells. *Inset*: dS separated by strandedness in Region 12 using the appropriate secondary structure. Lines indicate the median, and whiskers indicate the interquartile range. *P < 0.05 by equal variance unpaired Student’s *t* test. (*B*) RNA secondary structure of well-folded Region 11, which has identical nucleotide boundaries and nonidentical connectivity in Vero (*Left*) and C6/36 (*Right*) cells. (*C*) RNA secondary structure of well-folded Region 10, which has nonidentical nucleotide boundaries, but identical connectivity in the region shared in Vero (*Left*) and C6/36 (*Right*) cells.

In order to provide evolutionary support for well-folded RNA secondary structures, researchers studying positive-sense RNA viruses often analyze synonymous substitution rates (dS) (20, 50–53). This mode of analysis relies on the assumption that, if a secondary structure is evolutionarily conserved, synonymous mutations should accumulate more quickly at single-stranded regions relative to double-stranded. We therefore constructed an alignment of 54 mosquito-borne *flaviviral* ORFs and computed relative dS for well-folded regions in the WNV genome. We only observed significantly elevated dS for single-stranded codons relative to double-stranded codons for a single well-folded region (Fig. 5 *A*, *Inset*). This region, designated Region 12, is the largest well-folded region identified in either cell type. It contains two small stem-loop structures that flank a large stem capped with a multihelix junction, which are RNA motifs that have shown promise as drug targets (54). Importantly, the reactivity data from both cell types agree strongly with the predicted structure (Fig. 5*A*). As such, Region 12 represents an ideal target for functional validation.

However, as dS analysis flagged no other well-folded regions, we recognized the need to generate criteria to prioritize other regions for functional validation. Because we expected conserved riboregulatory elements to fold in both cell types, we reasoned the structural homology of well-folded regions between cell types may be used as a prioritization criteria. To that end, we performed SENS and PPV calculations using the Vero secondary structure of each well-folded region as the “predicted” structure, and the C6/36 structure as the “accepted” structure (Table 1). Using these results, we selected other targets for functional validation based on three different patterns of structural homology: identical nucleotide boundaries and near identical connectivity (Regions 8 and 12), identical boundaries and nonidentical connectivity (Regions 6 and 11), and nonidentical boundaries but identical connectivity (Region 10 and 16).

### Functional Validation of Candidate Structures by Targeted ASO LNA Disruption.

Targeted disruption of RNA secondary structures using ASO LNAs has emerged as a powerful tool to identify functional RNA structures in viral genomes (20, 50, 55). This method relies on the ability of LNAs, non-natural RNA base analogues that increase the T_m_ of a given duplex by 2 to 8 °C, to outcompete naturally occurring RNA–RNA duplexes (56). While previous studies used reporter constructs to measure viral growth, we instead generated a workflow to monitor their effects in this study. Specifically, we rely on cotransfection of in vitro transcribed, capped WNVic and ASO LNAs, with a qRT-PCR assay used to afford direct and highly accurate quantitation of WNV growth (43, 57). We validated our cotransfection system using an ASO LNA targeting the 3′CYC, 3′DAR-II, and 3′DAR-I cyclization elements to block genome cyclization. As expected, introduction of the cyclization ASO LNA resulted in a >3 log_10_-fold reduction in viral growth in both cell types compared to a nontargeting LNA (*SI Appendix*, Fig. S6 *A* and *B*; 3′CYC v nontargeting).

**Fig. 6.**
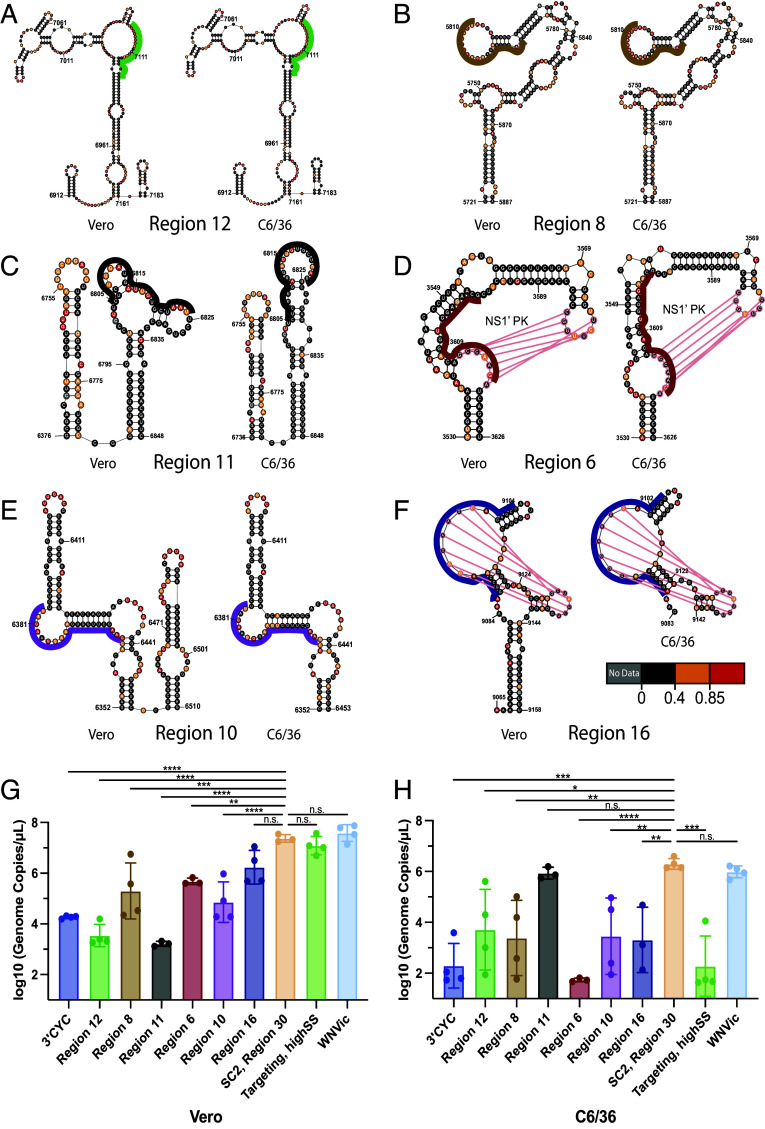
Targeted disruption of RNA structures with ASO LNAs results in potent viral growth defects. (*A*–*F*) Schematic showing LNAs (colored lines) targeted to well-folded regions determined in Vero (Left) or C6/36 (Right) cells. Region-specific LNAs are colored as in G and H. (*G* and *H*) Virus growth as measured by quantifying viral genomes in cell supernatant with qRT-PCR in *A* Vero cells at 3 dpi or (*B*) C6/36 cells at 6 dpi. Data points represent independent technical replicates. Bar height is the mean, and whiskers represent the SD. n.s., not significant; *P < 0.05, **P < 0.01, ***P < 0.001, and ****P < 0.0001 by ordinary one-way ANOVA with multiple comparisons.

Subsequently, we tested the six candidate ORF structures described above, prioritized for functional validation based on patterns of structural homology in different cellular contexts. All ASO LNAs were designed for maximal structure disruption, and they target both single- and double-stranded regions of the well-folded RNA Fig. 6 *C*–*H* structures (Fig. 6 *A*-*F*; colored lines). A list of all ASO LNAs used in this study is available in *SI Appendix*, Table S4.

Of the six ASO LNAs targeted to well-folded regions in the WNV ORF, four mediate significant defects in WNV growth in both cell types tested (Fig. 6 *G* and *H*). Indeed, some of the replication defects observed are more pronounced than the effect observed with the cyclization ASO LNA, suggesting that they mediate profound disruption of the viral life cycle. These include the ASO LNAs targeting Region 12 and Region 8, well-folded regions that share identical nucleotide boundaries and near-identical connectivity in both cell types (Fig. 6 *A* and *B*). Significant defects were also observed when targeting a substructure in Region 10 that appears with identical connectivity (100% SENS, 100% PPV) in both cell types, though the well-folded region is larger in Vero cells (Fig. 6*E*). Most interestingly, significant defects were observed when targeting Region 6, though the defect is ~3 log_10_-fold stronger in C6/36 cells. This region contains a previously reported PK in NS2A (NS1’ PK) that mediates production of a NS1 variant via programmed ribosomal (Fig. 6*D*; pink lines; *SI Appendix*, Fig. S2 *C* and *D*) (37, 58, 59).

Of the six ASO LNAs targeted to well-folded regions in the WNV ORF, two of them mediate significant defects in WNV growth in only a single cell type tested (Fig. 6 *G* and *H*). Specifically, Region 16, a region with nonidentical boundaries that contains a PK predicted in both cell types resulted in a significant growth defect in C6/36 cells only (Fig. 6*F*). Conversely, the ASO LNA targeting Region11 mediates a significant ~4 log_10_-fold reduction in WNV growth in Vero cells but has no effect on viral growth in C6/36 cells.

To provide an orthogonal method for evaluating the effects observed with ASO LNAs, we generated two mutant genomes that contain structure-disrupting synonymous mutation in either Region 11 or Region 12. Upon transfection into Vero cells, we observed significant defects in WNV growth in Vero cells (*SI Appendix*, Fig. S4*A*). These results suggest that the defects mediated by LNAs arise as a result of structure disruption, confirming via classical methods that these two regions play important functional roles in the viral life-cycle.

As all of the RNA structures described above were identified on the basis of their lowSS signature, we were interested to target a region that appears with highSS signatures in both cell types. To that end, we designed an ASO LNA against a region with Shannon entropy and SHAPE reactivity *above* the global median in both cell types (Genome coords. = 8920 to 8939). Interestingly, while this ASO LNA mediated no effect in Vero cells, we observed a significant ~4 log_10_-fold defect in C6/36 cells (Fig. 6 *G* and *H*). While prior work with SARS-CoV-2 showed that ASO LNAs targeted against highSS regions have no effect, these results suggests that highSS regions might harbor regulatory RNA sequence, though not necessarily RNA structure, that can play host-specific functional roles (20). Together, these data provide functional validation of the predicted secondary structures identified by SHAPE-MaP analysis, identify cell type–specific and conserved structures, and confirm that regions lacking well-supported structures may also harbor RNA sequences with functional roles.

## Discussion

It has long been known that the genomes of positive sense RNA viruses have the potential to form complex secondary and tertiary structures, but only recently has it become possible to apply high-throughput in vivo, genome-wide structure probing techniques to determine whether these structures persist and play a functional role within infected cells. It is of particular interest to monitor the genomic RNA structures of flaviviruses, as functional structures contained within the viral genome might have to persist as the virus alters between arthropod vector and either mammalian or bird hosts. If one could monitor the RNA secondary structure of a flavivirus in living cells, one might better understand the role of specific RNA structures in the virus lifecycle, determine whether different host cell environments can drive changes in genomic RNA structure and functions, and identify whether RNA structures might represent a promising class of drug targets against flaviviruses. While the secondary structures of flaviviruses such as Dengue and Zika have been examined in mammalian cell lines, no full-length flaviviral genome structure has been determined in arthropod vector context, and there has been no comparative study of viral RNA secondary structures between different hosts.

Here, we present the complete in-cell secondary structure of WNV, determined in cells from two different natural hosts: mosquito and primate. The resulting view of the entire genome in two different host contexts provides insight into poorly understood aspects of flaviviral biology, reveals unique roles for specific RNA motifs in the viral lifecycle, and underscores the persistence of viral genomic architecture in cells and across organisms. Importantly, the full-length structure for the WNV genome determined in mammalian cells allows us to make comparisons to the structures of other single-stranded, positive RNA viral genomes that have been probed or functionally evaluated in similar host contexts.

Overall, we find that the WNV genome has 54% BPC while actively replicating in mammalian cells, which is slightly less than reported for SARS-CoV-2 and Hepatitis C Virus (HCV) (56% and 60%, respectively) (20, 21). This is consistent with other work showing that SARS-CoV-2 and HCV have higher structure propensity than WNV (19). When considering the well-folded RNA content of WNV and other single-stranded, positive-sense viral RNA genomes, including SARS-CoV-2, HCV, chikungunya virus, Zika virus, and dengue virus, similarities abound. At the highest organizational level, these viruses contain regions of well-folded RNA dispersed throughout the genome, separated by relatively unstructured regions characterized by long stretches of high SHAPE reactivity, Shannon entropy, or both (20, 21, 50, 60, 61). While well-folded RNA content is found throughout the genome, it is not equivalently dispersed among individual domains. For example, the 5′UTR of SARS-CoV-2 is entirely folded into stable secondary structures, whereas another domain, encoding protein Nsp10, contains no well-folded RNA (20).

A similar pattern is observed in the mammalian WNV genome structure reported here; 100% of the nucleotides in the 5′UTR are defined as well-folded, while protein domain NS2B contains no well-folded RNA. In total, the mammalian WNV genome structure we have elucidated contains 19 well-folded regions, which is on par with other viruses with ~10 kb genomes (HCV = 15; chikungunya = 23; dengue = 16; Zika = 12) (21, 60, 61). Taken together, these shared architectural features may represent general organizing principles of positive sense RNA viral genomes, reflecting the competing requirements of protected, well-folded functional RNA structure with a requirement for service as a template for translation and replication.

Here, we report the first in-cell secondary structure of WNV in an arthropod cell line, which allows us to compare the WNV viral genomic structure in two different hosts. Overall, we observe a minimal host dependence of the WNV genome structure. This is reflected both in the strong correlations of SHAPE-MaP data as well as almost identical levels of base-pairing retained across the WNV genome regardless of cellular context. Furthermore, there is remarkable agreement in the location, size, and connectivity of well-folded regions within the genome (Table 1). These data suggest that much like proteins, RNA structure is encoded by the primary nucleic acid sequence. This general feature of RNA structure may confer durable fitness advantages to enzootic viruses as they alternate between hosts, and may provide further insight into the slow evolutionary rate of vector-borne viruses, thought to arise due to host-specific evolutionary pressures experienced by the virus as it replicates within a single host as well as genetic bottlenecks encountered as the virus switches hosts (62–64).

Both the WNV SHAPE-Map data and the resulting experimentally determined secondary structures from the two host contexts provide additional insights into previously studied but poorly understood aspects of flaviviral biology. Specifically, our work demonstrates that the linear conformation of the genomic RNA dominates in vivo and that the cyclized genome is dominant under the protein-free conditions used to study RNA in vitro. While several studies have identified protein-binding partners as promoters of genome cyclization, our data suggest that host factors, such as actively translating ribosomes, may push the genome away from its thermodynamically preferred cyclized conformation (12, 65). Importantly, sequestration of the genome away from host factors inside replication complexes might allow the genome to naturally cyclize. In this model, the “molecular switch” that mediates genome cyclization would be the formation of replication complexes, a process itself known to be dependent on the translation and accumulation of sufficient levels of WNV nonstructural proteins (47). This agrees with studies showing that alterations in the balance of linear and cyclized genomes can negatively impact viral replication kinetics, representing an elegant strategy for sequential timing of the viral life cycle in any host context (13, 14).

The structural maps we have obtained for the WNV 3′ viral terminus reveal a tripartite domain architecture, with each domain displaying a unique structural profile. Importantly, the amplicon design strategy ensures that SHAPE data are collected exclusively on the full-length genome, as even the largest sfRNA species is too small to be captured by the primers chosen for amplicon generation. Overall, the 3′ terminus includes a flexible, single-stranded region (Domain II) sandwiched between two highly structured, well-determined domains (Domains I and III). As Domain III corresponds to the largest sfRNA produced during the course of WNV infection, its position immediately downstream of Domain II may be important for stalling of Xrn1 at the appropriate position. Of additional note is a long-range base-pairing interaction that forms between Domain I and the 5′ end of Domain III, resulting in the formation of previously unreported long-range duplexes within Domain III. While this represents a deviation from canonical depictions of sfRNA structure, the structure we have obtained may simply represent a genome-specific fold of the 3′UTR (5, 7, 35). Our lab has previously highlighted the importance of upstream sequence context in accurate prediction of viral RNA structure (19). As the long-range duplexes we have identified have high Shannon entropy, it is likely that liberation of the smaller sfRNA species may be linked with local refolding of the DBI PK that occurs after Xrn1 has chewed through upstream duplexes.

Analysis of the in-cell genome structures enables the identification of potential riboregulatory elements within the WNV genome. Due to weak signals of RNA secondary structure conservation, we instead relied on an “empirical structure first” approach, using patterns of structural homology of well-folded regions between cell types to identify structures that may mediate conserved functional roles. With this strategy, we delineate three unique patterns of structural homology that allow us to prioritize six well-folded regions for functional validation.

Specifically, we identified four well-folded RNA structures in the WNV ORF that, upon disruption, result in severe growth defects in both cell types tested. Of these, three exhibit growth defects of comparable magnitude in both cell types. These regions would be interesting targets for follow-up studies using RNA antisense purification coupled with mass spectrometry (RAP–MS) methodologies (66). Of special interest, however, is Region 6 which contains the NS1’ PK. Careful molecular virology has confirmed production of an extended NS1 variant, and the in vivo reactivities we observe provide direct confirmation that the WNV NS1’ PK folds inside infected cells (*SI Appendix*, Fig. S2 *C* and *D*) (58). As NS1’ PK disruption was shown to attenuate WNV replication in live mosquitos and birds, our data suggest that the NS1’ PK ASO LNA may have applications as an antiviral (37). Similarly, the ASO LNA that blocks genome cyclization (3′CYC) and impairs replication might also prove efficacious as a pan-*flaviviral* therapeutic agent.

We also identified two well-folded RNA structures within the WNV ORF that, upon disruption, mediate cell type–specific growth defects. To date, functional RNA structures that mediate host-specific effects in flaviviruses have only been identified in regions proximal to viral termini, including UTRs and the capsid ORF (26, 67). Since functional RNAs are often associated with protein binding, the observed host-specific function of these two regions may reflect the recruitment of host-specific proteins, and therefore represent ideal targets for follow-up RAP–MS studies. The data for Region 11, however, suggest a more interesting mechanistic explanation. If the profound (~4 log_10_-fold) defect observed in Vero cells was attributable to protein binding, one would expect lower reactivities in this region in Vero cells because protein binding is known to occlude access of SHAPE reagents (48). However, the opposite is true; the region targeted by the ASO LNA contains three contiguous nucleotides that are *more* highly reactive in Vero cells. As Vero cells are cultured at a higher temperature than C6/36 cells (37 °C vs. 28 °C, respectively), one possible explanation for the enhanced reactivity of these nucleotides may be due to RNA unfolding. The presence of a functionally important, thermally responsive RNA structure may suggest the WNV genome harbors an RNA thermometer, which are host-sensing elements first identified in pathogenic bacteria (24). While prior work has pointed to the base of 3′SL in the WNV 3′UTR as a thermally responsive element that influences genome cyclization, Region 11 represents the first thermally responsive element identified in a viral ORF (27). However, more work is required to establish a conclusive link between the observed structural and functional differences and temperature.

The work presented here deepens our understanding of the WNV life cycle, revealing a global genome fold with minimal host dependence. We show that the linear genome conformation dominates within infected cells, and we propose the formation of replication complexes as the molecular switch that allows the virus to transition from early translation to late-stage replication. We also reveal a tripartite domain architecture of the 3′UTR that may provide mechanistic insights into sfRNA production. We identify six novel riboregulatory elements, a few of which appear to play host-specific functional roles. Our study also demonstrates that patterns of structural homology can serve as powerful indicators of functional RNA structure, a method that can be readily extended to other enzootic viruses. Finally, the identification of ASO LNAs that induce highly specific, potent defects in WNV growth represents an exciting development in the field of antiviral nucleic acid therapeutics.

## Supplementary Material

Appendix 01 (PDF)

## Data Availability

SHAPE-MaP sequencing data have been deposited in GEO Accession Database (GSE228446) (46).

## References

[1] J. Whitehorn, S. Yacoub, Global warming and arboviral infections. Clin. Med. J. R. Coll. Phys. Lond. **19**, 149–152 (2019). 10.7861/clinmedicine.19-2-149 PMC6454362 30872300

[2] E. Lee , Structures of flavivirus RNA promoters suggest two binding modes with NS5 polymerase. Nat. Commun. **12**, 1–12 (2021). 33953197 10.1038/s41467-021-22846-1 PMC8100141

[3] K. H. Choi, The role of the stem-loop a rna promoter in flavivirus replication. Viruses **13**, 1107 (2021). 34207869 10.3390/v13061107 PMC8226660

[4] H. Dong, B. Zhang, P. Y. Shi, Terminal structures of West Nile virus genomic RNA and their interactions with viral NS5 protein. Virology **381**, 123–135 (2008). 18799181 10.1016/j.virol.2008.07.040

[5] A. MacFadden , Mechanism and structural diversity of exoribonuclease-resistant RNA structures in flaviviral RNAs. Nat. Commun. **9**, 1–11 (2018). 29317714 10.1038/s41467-017-02604-y PMC5760640

[6] G. P. Göertz , Noncoding subgenomic Flavivirus RNA is processed by the mosquito RNA interference machinery and determines West Nile Virus transmission by culex pipiens mosquitoes. J. Virol. **90**, 10145–10159 (2016). 27581979 10.1128/JVI.00930-16 PMC5105652

[7] G. P. Pijlman , A highly structured, nuclease-resistant, noncoding RNA produced by flaviviruses is required for pathogenicity. Cell Host Microbe **4**, 579–591 (2008). 19064258 10.1016/j.chom.2008.10.007

[8] M. Basu, M. A. Brinton, West Nile virus (WNV) genome RNAs with up to three adjacent mutations that disrupt long distance 5′-3′ cyclization sequence basepairs are viable. Virology **412**, 220–232 (2011). 21292293 10.1016/j.virol.2011.01.008 PMC3056923

[9] B. Zhang, H. Dong, D. A. Stein, P. L. Iversen, P. Shi, West Nile virus genome cyclization and RNA replication require two pairs of long-distance RNA interactions. Virology **373**, 1–13 (2008). 18258275 10.1016/j.virol.2008.01.016

[10] R. Suzuki, R. Fayzulin, I. Frolov, P. W. Mason, Identification of mutated cyclization sequences that permit efficient replication of West Nile virus genomes: Use in safer propagation of a novel vaccine candidate. J. Virol. **82**, 6942–51 (2008). 18480453 10.1128/JVI.00662-08 PMC2446964

[11] P. Friebe, P. Shi, E. Harris, The 5’ and 3’ downstream AUG region elements are required for mosquito-borne Flavivirus RNA replication. J. Virol. **85**, 1900–1905 (2011). 21123391 10.1128/JVI.02037-10 PMC3028882

[12] W. G. Davis, M. Basu, E. J. Elrod, M. W. Germann, M. A. Brinton, Identification of cis-acting nucleotides and a structural feature in West Nile Virus 3-terminus RNA that facilitate viral minus strand. J. Virol. **87**, 7622–7636 (2013). 23637406 10.1128/JVI.00212-13 PMC3700269

[13] S. M. Villordo, D. E. Alvarez, A. V. Gamarnik, A balance between circular and linear forms of the dengue virus genome is crucial for viral replication. RNA **16**, 2325–2335 (2010). 20980673 10.1261/rna.2120410 PMC2995394

[14] Z. Liu, X. Li, T. Jiang, Y. Deng, Q. Ye, Viral RNA switch mediates the dynamic control of flavivirus replicase recruitment by genome cyclization. Elife **5**, e17636 (2016), 10.7554/eLife.17636. 27692070 PMC5101012

[15] N. G. Iglesias, A. V. Gamarnik, Dynamic RNA structures in the dengue virus genome. RNA Biol. **8**, 249–257 (2011). 21593583 10.4161/rna.8.2.14992

[16] L. M. Simon , In vivo analysis of influenza A mRNA secondary structures identifies critical regulatory motifs. Nucleic Acids Res. **47**, 7003–7017 (2019). 31053845 10.1093/nar/gkz318 PMC6648356

[17] S. Rouskin, M. Zubradt, S. Washietl, M. Kellis, J. S. Weissman, Genome-wide probing of RNA structure reveals active unfolding of mRNA structures in vivo. Nature **505**, 701–705 (2014). 24336214 10.1038/nature12894 PMC3966492

[18] P. Li , Integrative analysis of Zika virus genome RNA structure reveals critical determinants of viral infectivity. Cell Host Microbe **24**, 875–886.e5 (2018). 30472207 10.1016/j.chom.2018.10.011

[19] R. d. C. A. Tavares, G. Mahadeshwar, H. Wan, N. C. Huston, A. M. Pyle, The global and local distribution of RNA structure throughout the SARS-CoV-2 genome. J. Virol. **95**, 1–17 (2021). 10.1128/JVI.02190-20 PMC8092842 33268519

[20] N. C. Huston , Comprehensive in-vivo secondary structure of the SARS-CoV-2 genome reveals novel regulatory motifs and mechanisms. Mol. Cell **81**, 1–15 (2021). 33444546 10.1016/j.molcel.2020.12.041 PMC7775661

[21] H. Wan, R. L. Adams, B. D. Lindenbach, A. M. Pyle, The in vivo and in vitro architecture of the Hepatitis C virus RNA genome uncovers functional RNA secondary and tertiary structures. J. Virol. **96**, 1–23 (2022). 10.1128/jvi.01946-21 PMC9044954 35353000

[22] E. Kikovska, S. G. Svärd, L. A. Kirsebom, Eukaryotic RNase P RNA mediates cleavage in the absence of protein. Proc. Natl. Acad. Sci. U.S.A. **104**, 2062–2067 (2007). 17284611 10.1073/pnas.0607326104 PMC1892975

[23] A. M. Pyle, Metal ions in the structure and function of RNA. J. Biol. Inorg. Chem. **7**, 679–690 (2002). 12203005 10.1007/s00775-002-0387-6

[24] J. Kortmann, F. Narberhaus, Bacterial RNA thermometers: Molecular zippers and switches. Nat. Publ. Gr. **10**, 255–265 (2012). 10.1038/nrmicro2730 22421878

[25] S. M. Villordo, C. V. Filomatori, I. Sánchez-vargas, C. D. Blair, Dengue virus RNA structure specialization facilitates host adaptation. PLoS Pathog. **11**, 1–22 (2015). 10.1371/journal.ppat.1004604 PMC4311971 25635835

[26] L. de Borba , RNA structure duplication in the Dengue virus 3’ UTR: Redundancy or host specificity? MBio **10**, 1–18 (2019). 10.1128/mBio.02506-18 PMC6325252 30622191

[27] A. Meyer , An RNA thermometer activity of the West Nile virus genomic 30-terminal stem-loop element modulates viral replication efficiency during host switching. Viruses **12**, 1–22 (2020). 10.3390/v12010104 PMC7019923 31952291

[28] N. A. Siegfried, S. Busan, G. M. Rice, J. A. E. Nelson, K. M. Weeks, RNA motif discovery by SHAPE and mutational profiling (SHAPE-MaP). Nat. Methods **11**, 959–965 (2014). 25028896 10.1038/nmeth.3029 PMC4259394

[29] G. Q. Tang , Relaxed rotational and scrunching changes in P266L mutant of T7 RNA polymerase reduce short abortive RNAs while delaying transition into elongation. PLoS One **9**, 1–12 (2014). 10.1371/journal.pone.0091859 PMC3961267 24651161

[30] R. L. Adams, N. C. Huston, R. C. A. Tavares, A. M. Pyle, Sensitive detection of structural features and rearrangements in long, structured RNA molecules. Methods Enzymol. **623**, 249–289 (2019). 31239050 10.1016/bs.mie.2019.04.002

[31] D. J. Korbie, J. S. Mattick, Touchdown PCR for increased specificity and sensitivity in PCR amplification. Nat. Protoc. **3**, 1452–1456 (2008). 18772872 10.1038/nprot.2008.133

[32] S. Busan, K. M. Weeks, Accurate detection of chemical modifications in RNA by mutational profiling (MaP) with ShapeMapper 2. RNA **24**, 143–148 (2018). 29114018 10.1261/rna.061945.117 PMC5769742

[33] C. E. Hajdin , Accurate SHAPE-directed RNA secondary structure modeling, including pseudoknots. Proc. Natl. Acad. Sci. U.S.A. **110**, 5498–5503 (2013). 23503844 10.1073/pnas.1219988110 PMC3619282

[34] G. Faggioni , West Nile alternative open reading frame (N-NS4B/WARF4) is produced in infected West Nile Virus (WNV) cells and induces humoral response in WNV infected individuals. Virol. J. **9**, 1–14 (2012). 23173701 10.1186/1743-422X-9-283 PMC3574045

[35] A. Funk , RNA structures required for production of subgenomic flavivirus RNA. J. Virol. **84**, 11407–11417 (2010). 20719943 10.1128/JVI.01159-10 PMC2953152

[36] M. J. Smola, G. M. Rice, S. Busan, N. A. Siegfried, K. M. Weeks, Selective 2’-hydroxyl acylation analyzed by primer extension and mutational profiling (SHAPE-MaP) for direct, versatile and accurate RNA structure analysis. Nat. Protoc. **10**, 1643–1669 (2015). 26426499 10.1038/nprot.2015.103 PMC4900152

[37] E. B. Melian , Programmed ribosomal frameshift alters expression of West Nile Virus genes and facilitates virus replication in birds and mosquitoes. PLoS Pathog. **10**, e1004447 (2014). 25375107 10.1371/journal.ppat.1004447 PMC4223154

[38] S. Bellaousov, J. S. Reuter, M. G. Seetin, D. H. Mathews, RNAstructure: Web servers for RNA secondary structure prediction and analysis. Nucleic Acids Res. **41**, W471–W474 (2010). 10.1093/nar/gkt290 PMC3692136 23620284

[39] G. Moureau , New insights into Flavivirus evolution, taxonomy and biogeographic history, extended by analysis of canonical and alternative coding sequences. PLoS One **10**, e0117849 (2015). 25719412 10.1371/journal.pone.0117849 PMC4342338

[40] V. Ranwez, E. J. P. Douzery, C. Cambon, N. Chantret, F. Delsuc, MACSE v2: Toolkit for the alignment of coding sequences accounting for frameshifts and stop codons. Mol. Biol. Evol. **35**, 2582–2584 (2018). 30165589 10.1093/molbev/msy159 PMC6188553

[41] A. M. Waterhouse, J. B. Procter, D. M. A. Martin, M. Clamp, G. J. Barton, Jalview Version 2-A multiple sequence alignment editor and analysis workbench. Bioinformatics **25**, 1189–1191 (2009). 19151095 10.1093/bioinformatics/btp033 PMC2672624

[42] B. Murrell , FUBAR: A fast, unconstrained bayesian AppRoximation for inferring selection. Mol. Biol. Evol. **30**, 1196–1205 (2013). 23420840 10.1093/molbev/mst030 PMC3670733

[43] R. S. Lanciotti , Rapid detection of West Nile Virus from human clinical specimens. J. Clin. Pathol. **38**, 4066–4071 (2000). 10.1128/jcm.38.11.4066-4071.2000 PMC87542 11060069

[44] P. Shi, M. Tilgner, M. K. Lo, K. A. Kent, K. A. Bernard, Infectious cDNA clone of the epidemic West Nile Virus from New York City. J. Virol. **76**, 5847–5856 (2002). 12021317 10.1128/JVI.76.12.5847-5856.2002 PMC136194

[45] E. J. Merino, K. A. Wilkinson, J. L. Coughlan, K. M. Weeks, RNA structure analysis at single nucleotide resolution by selective 2′-hydroxyl acylation and primer extension (SHAPE). J. Am. Chem. Soc. **127**, 4223–4231 (2005), 10.1021/ja043822v. 15783204

[46] N. C. Huston, L. H. Tsao, D. E. Brackney, A. M. Pyle, West Nile virus genome harbors essential riboregulatory elements with conserved and host-specific functional roles. NCBI GEO. https://www.ncbi.nlm.nih.gov/geo/query/acc.cgi?acc=GSE228446. Deposited 28 March 2023. 10.1073/pnas.2312080121 PMC11260092 38985757

[47] M. A. Brinton, Replication cycle and molecular biology of the West Nile Virus. Viruses **6**, 13–53 (2014), 10.3390/v6010013. PMC3917430 24378320

[48] M. J. Smola, J. M. Calabrese, K. M. Weeks, Detection of RNA-protein interactions in living cells with SHAPE. Biochemistry **54**, 6867–6875 (2015). 26544910 10.1021/acs.biochem.5b00977 PMC4900165

[49] C. Polacek, P. Friebe, E. Harris, Poly(A)-binding protein binds to the non-polyadenylated 3’ untranslated region of dengue virus and modulates translation efficiency. J. Gen. Virol. **90**, 687–692 (2009). 19218215 10.1099/vir.0.007021-0

[50] E. A. Dethoff , Pervasive tertiary structures in the Dengue virus RNA genome modulate fitness. Proc. Natl. Acad. Sci. **115**, 11513–11518 (2018). 30341219 10.1073/pnas.1716689115 PMC6233125

[51] A. Tuplin, J. Wood, D. J. Evans, A. H. Patel, P. Simmonds, Thermodynamic and phylogenetic prediction of RNA secondary structures in the coding region of hepatitis C virus. RNA **8**, 824–841 (2002). 12088154 10.1017/s1355838202554066 PMC1370300

[52] R. Assis, Strong epistatic selection on the RNA secondary structure of HIV. PLoS Pathog. **10**, e1004363 (2014). 25210786 10.1371/journal.ppat.1004363 PMC4161434

[53] P. Simmonds, D. B. Smith, Structural constraints on RNA virus evolution. J. Virol. **73**, 5787–5794 (1999). 10364330 10.1128/jvi.73.7.5787-5794.1999 PMC112639

[54] K. D. Warner, C. E. Hajdin, K. M. Weeks, Principles for targeting RNA with drug-like small molecules. Nat. Rev. Drug Discov. **17**, 547–558 (2018). 29977051 10.1038/nrd.2018.93 PMC6420209

[55] A. Tuplin, M. Struthers, J. Cook, K. Bentley, D. J. Evans, Inhibition of HCV translation by disrupting the structure and interactions of the viral CRE and 3’ X-tail. Nucleic Acids Res. **43**, 2914–2926 (2015). 25712095 10.1093/nar/gkv142 PMC4357731

[56] K. E. Lundin , Biological activity and biotechnological aspects of locked nucleic acids. Adv. Genet. **82**, 47–107 (2013). 23721720 10.1016/B978-0-12-407676-1.00002-0

[57] D. E. Brackney, J. E. Beane, G. D. Ebel, RNAi targeting of West Nile virus in mosquito midguts promotes virus diversification. PLoS Pathog. **5**, 1–9 (2009). 10.1371/journal.ppat.1000502 PMC2698148 19578437

[58] E. B. Melian , NS1’ of Flaviviruses in the Japanese encephalitis virus serogroup is a product of ribosomal frameshifting and plays a role in viral neuroinvasiveness. J Virol. **84**, 1641–1647 (2010). 19906906 10.1128/JVI.01979-09 PMC2812330

[59] E. R. Winkelmann, D. G. Widman, R. Suzuki, P. W. Mason, Analyses of mutations selected by passaging a chimeric flavivirus identify mutations that alter infectivity and reveal an interaction between the structural proteins and the nonstructural glycoprotein NS1. Virology **421**, 96–104 (2011). 21999990 10.1016/j.virol.2011.09.007

[60] R. G. Huber , Structure mapping of dengue and Zika viruses reveals functional long-range interactions. Nat. Commun. **10**, 1408 (2019). 30926818 10.1038/s41467-019-09391-8 PMC6441010

[61] E. A. Madden , Using SHAPE-MaP to model rna secondary structure and identify 3′UTR variation in Chikungunya Virus. J. Virol. **94**, e00701-20 (2020). 32999019 10.1128/JVI.00701-20 PMC7925192

[62] C. H. Woelk, E. C. Holmes, Reduced positive selection in vector-borne RNA viruses. Mol. Biol. Evol. **19**, 2333–2336 (1998). 10.1093/oxfordjournals.molbev.a004059 12446826

[63] N. D. Grubaugh , Experimental evolution of an RNA virus in wild birds: Evidence for host-dependent impacts on population structure and competitive fitness. PLoS Pathog. **11**, e1004874 (2015), 10.1371/journal.ppat.1004874. 25993022 PMC4439088

[64] N. D. Grubaugh, G. D. Ebel, Dynamics of West Nile virus evolution in mosquito vectors. Curr. Opin. Virol. **21**, 132–138 (2016). 27788400 10.1016/j.coviro.2016.09.007 PMC5384794

[65] J. L. Blackwell, M. A. Brinton, Translation elongation factor-1 alpha interacts with the 3 J stem-loop region of West Nile Virus genomic RNA. J. Virol. **71**, 6433–6444 (1997). 9261361 10.1128/jvi.71.9.6433-6444.1997 PMC191917

[66] C. A. McHugh , The Xist lncRNA interacts directly with SHARP to silence transcription through HDAC3. Nature **521**, 232–236 (2015). 25915022 10.1038/nature14443 PMC4516396

[67] S. M. Villordo, A. V. Gamarnik, Differential RNA sequence requirement for Dengue virus replication in mosquito and mammalian cells. J. Virol. **87**, 9365–9372 (2013). 23760236 10.1128/JVI.00567-13 PMC3754043

