# Supplementary material for: The West Nile virus genome harbors essential riboregulatory elements with conserved and host-specific functional roles
Source: Proc Natl Acad Sci U S A. 2024 Jul 10;121(29):e2312080121. doi: 10.1073/pnas.2312080121 (PMC11260092; doi:10.1073/pnas.2312080121)
Supplement: Supplementary file 1 — Appendix 01 (PDF) [file pnas.2312080121.sapp.pdf]

PNAS

Supporting Information for
West Nile virus genome harbors essential riboregulatory elements with conserved and host-specific functional roles

Nicholas C Huston,[a] Lucille H. Tsao,[d] Doug E Brackney,[b] Anna Marie Pyle[c,d,e,#]

#Address correspondence to Anna Marie Pyle
Email: anna.pyle@yale.edu

This PDF file includes:

Figures S1 to S4
Tables S1 to S5

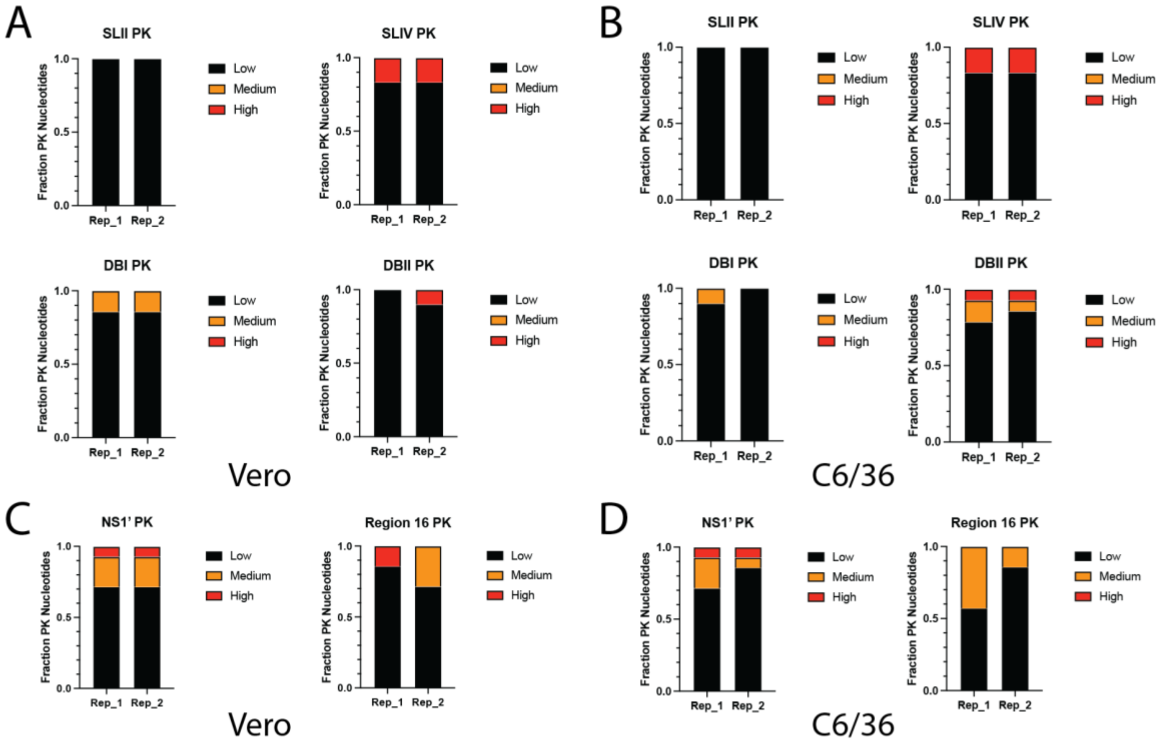

Fig. S1. Analysis of SHAPE-MaP reactivities of pseudoknotted nucleotides in the WNV genome confirms the formation of these pseudoknots *in vivo*. A) Nucleotides of each pseudoknot in the WNV 3'UTR, binned by normalized reactivity collected in Vero cells, with bin size expressed as a fraction of total nucleotides in that pseudoknot. B) Nucleotides of each pseudoknot in the WNV 3'UTR, binned by normalized reactivity collected in C6/36 cells, plotted as in (A). C) Nucleotides of the NS1' pseudoknot (left) or a novel pseudoknot predicted to fold in Region 16 (right), binned by normalized reactivity collected in Vero cells, plotted as in (A). D) Nucleotides of the NS1' pseudoknot (left) or a novel pseudoknot predicted to fold in Region 16 (right), binned by normalized reactivity collected in C6/36 cells, plotted as in (A).

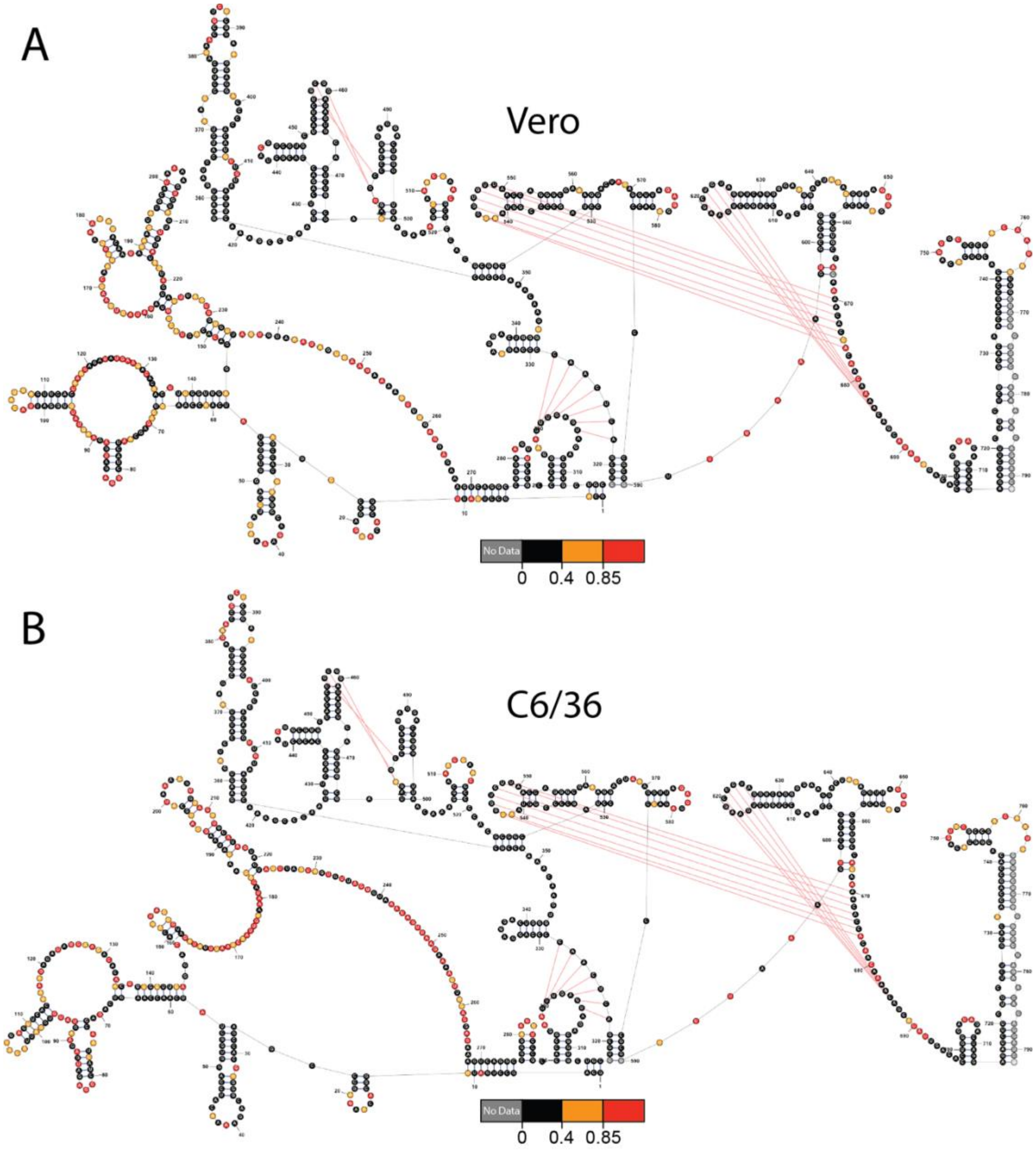

Fig. S2. Normalized SHAPE reactivity mapped to the structure prediction of the 3' viral terminus reveals domain-specific patterns of RNA backbone flexibility Structure of the 3' viral terminus determined in infected (A) Vero cells or (B) C6/36, color-coded by normalized SHAPE reactivity.

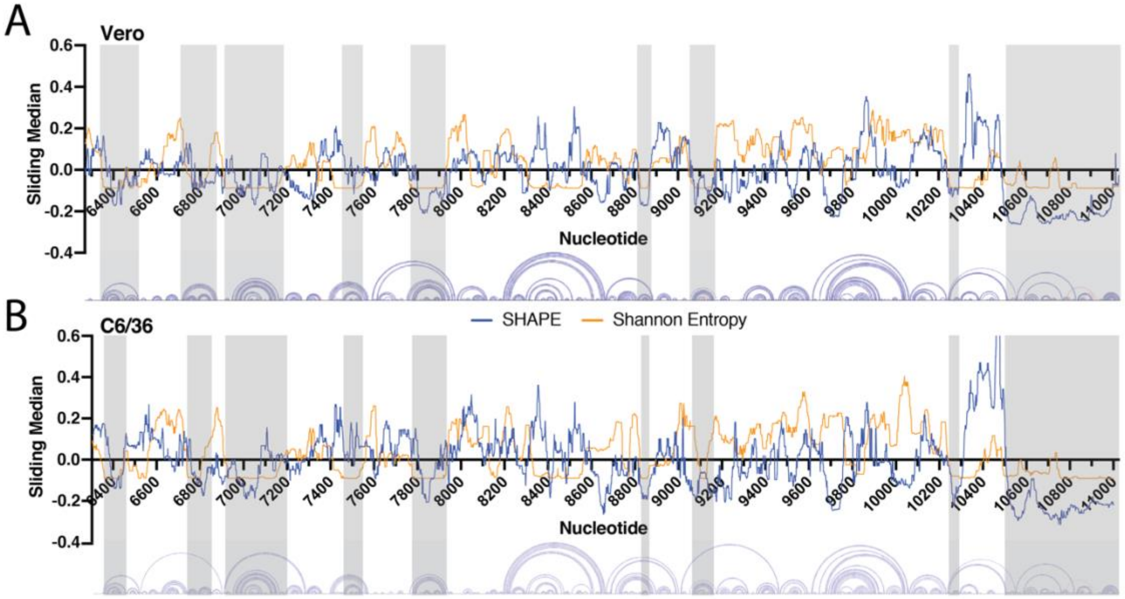

Figure S3. West Nile Virus genome folds into networks of well-folded regions with little apparent host dependency Analysis of SHAPE reactivities and Shannon entropy reveals the presence of highly structured, well-determined domains in the second half of the WNV genome in A) Vero cells or B) C6/46 cells. Nucleotide coordinates are indicated on the x-axis. Local median SHAPE reactivity and Shannon entropy are indicated by blue and orange lines, respectively. Well-folded regions that appear in both or only a single cell type are shaded with gray or red boxes, respectively. Arc plots for predicted base-pairing interactions in the structural model are shown below the x-axis.

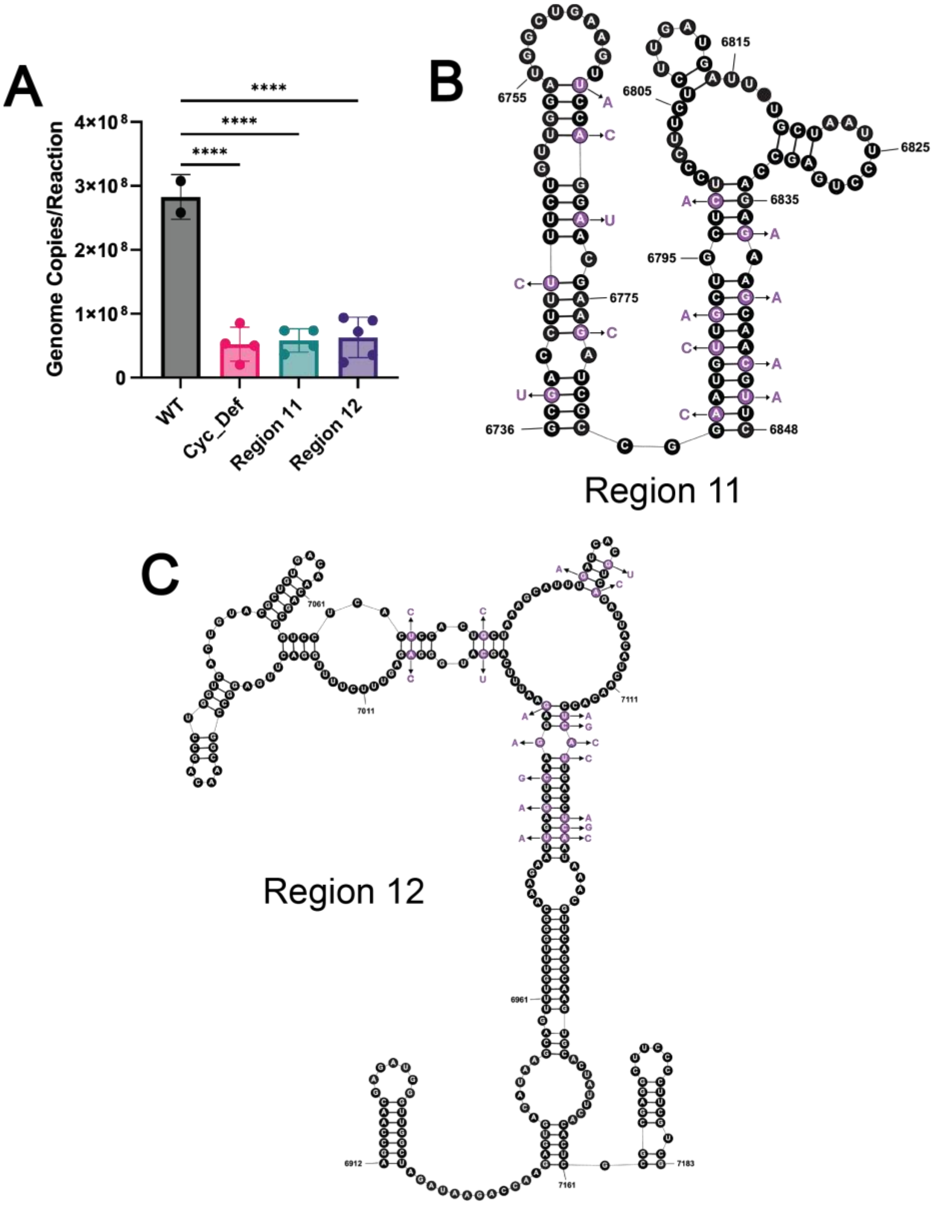

Figure S4 Targeted disruption of RNA structures with mutagenesis results in viral growth defects A) Virus growth as measured by quantifying viral genomes in cell supernatant with qRT-PCR in Vero cells at 7dpi. Data points represent independent technical replicates. Bar height is the mean, and whiskers represent standard deviation. WT = wild-type; Cyc_Def = cyclization defective. ****$p < 0.0001$ by ordinary one-way ANOVA with multiple comparisons. B/C) Nucleotides that were mutated for targeted disruption of Region 11 (B) and Region 12 (C) are highlighted in purple with an arrow pointing to what the nucleotide was mutated to.

Table S1. Primers used in this study

Primer Name	Sequence	Purpose
Cyc_Def_F	aaGACACCTGGGATAGACTAGG	Cyc_Defect_F
Cyc_Def_R	ataGCTGTTTTGTTGTGGTGTTTTG	Cyc_Defect_R
WNV_1160_F	TCAGCGATCTCTCCACCAAAG	for TaqMan Assay
WNV_1229_R	GGGTCAGCACGTTTGTCATTG	for TaqMan Assay
WNV_1031_F	TAATACGACTCACTATAGATTTGGTTCTCGAAGGCGACAG	qRT-PCR Standard Generation
WNV_3430_R	GTGGTGGTAAGGTGCAGCTC	qRT-PCR Standard Generation
F_PCR_WNV_Amplicon_1	GAAGTATGTGGATTACATGAGTTCA	SHAPE-MaP
F_PCR_WNV_Amplicon_2	AGGAAAACGAGAGGACATC	SHAPE-MaP
F_PCR_WNV_Amplicon_3	CTTCCTCAATGCTATGTCA	SHAPE-MaP
F_PCR_WNV_Amplicon_4	AGAGAGAGAAAAAACCCGG	SHAPE-MaP
F_PCR_WNV_Amplicon_5	TCAGTGAATATGACCAGCC	SHAPE-MaP
F_PCR_WNV_Amplicon_6	ATAACATGGACACTCATAAAGAACA	SHAPE-MaP
F_PCR_WNV_Amplicon_7	GGGAGAGTTTCTTTTGGAC	SHAPE-MaP
F_PCR_WNV_Amplicon_8	CGAAGCTTGGTGAAAGGAA	SHAPE-MaP
F_PCR_WNV_Amplicon_9	AAGTAGTCCAATTGAACAGAAAGTC	SHAPE-MaP
F_PCR_WNV_Amplicon_10	TAGTGCAGGGTGAAAGGAT	SHAPE-MaP
F_PCR_WNV_Amplicon_11	GGAGCACCTTGGAAGATAT	SHAPE-MaP
F_PCR_WNV_Amplicon_12	AATGGCTTATCACGATGCC	SHAPE-MaP
F_PCR_WNV_Amplicon_13	CTGGGTACAAGACACAAAA	SHAPE-MaP
F_PCR_WNV_Amplicon_14	GGAAGCAGTGAAGGACGAG	SHAPE-MaP
F_PCR_WNV_Amplicon_15	TTGGTCACTGTCAACCCTT	SHAPE-MaP
F_PCR_WNV_Amplicon_16	AACTACTCCACACAGGTTG	SHAPE-MaP
F_PCR_WNV_Amplicon_17	CACTGACAGTGCAGACACA	SHAPE-MaP
F_PCR_WNV_Amplicon_18	CTCGATGTCTAAGAAACCA	SHAPE-MaP
F_PCR_WNV_Amplicon_19	AGTAGTTCGCCTGTGTGAGCT	SHAPE-MaP
R_PCR_WNV_Amplicon_1	TCCTGTGTTCTCGCACCAC	SHAPE-MaP
R_PCR_WNV_Amplicon_2	AGATCTCCTAGTCTATCCCAG	SHAPE-MaP
R_PCR_WNV_Amplicon_3	CTATAAAACTACACTTTTATGCATA	SHAPE-MaP
R_PCR_WNV_Amplicon_4	TCATGATCAATTCAGTGAAAT	SHAPE-MaP
R_PCR_WNV_Amplicon_5	GAGTTCTTTCTTCCAAGCCAG	SHAPE-MaP
R_PCR_WNV_Amplicon_6	ACATCTTCCTCGTATTGGGGT	SHAPE-MaP
R_PCR_WNV_Amplicon_7	TTCTTTCCAAACCTCTCCCAA	SHAPE-MaP
R_PCR_WNV_Amplicon_8	TTATTGAGGTCAATGAGGTGT	SHAPE-MaP
R_PCR_WNV_Amplicon_9	ATCTGAGAACGTTTTCCCGAG	SHAPE-MaP
R_PCR_WNV_Amplicon_10	TTCAGATATGTCTGTTGTGAT	SHAPE-MaP
R_PCR_WNV_Amplicon_11	CAGTCTTCTGTTTATGGCCTC	SHAPE-MaP
R_PCR_WNV_Amplicon_12	TATCCAAAATCCAACTACTGA	SHAPE-MaP
R_PCR_WNV_Amplicon_13	ATGGCTCTCAGTATCATCCAA	SHAPE-MaP
R_PCR_WNV_Amplicon_14	TATCAACTTTCCGCTCTCTGT	SHAPE-MaP

R_PCR_WNV_Amplicon_15	TTAGGTGCTGACTTGTACATT	SHAPE-MaP
R_PCR_WNV_Amplicon_16	TGATCTGTTGTTCTCCTCTGC	SHAPE-MaP
R_PCR_WNV_Amplicon_17	ATTCTCCAAGCTTTAGTGTGT	SHAPE-MaP
R_PCR_WNV_Amplicon_18	CTGTTTTTACCAAATACCTTG	SHAPE-MaP
R_PCR_WNV_Amplicon_19	GTGCATCTTCCATACCTGA	SHAPE-MaP

Table S2. Aggregated quality control metrics from ShapeMapper 2.0

	Read Depth Check (%)	Mutation Rate Check (%)	High Background Check (%)	No. Highly Reactive (%)
**ShapeMapper Threshold**	**>80%**	**>50%**	**<5%**	**>8%**
**Vero, Rep1**	99.8 (11005/11029)	84.9 (9340/11005)	0.3 (35/11005)	15.7 (1723/11005)
**Vero, Rep2**	99.8 (11003/11029)	84.6 (9309/11003)	0.3 (35/11003)	10.1 (1107/1005)
**C6/36, Rep1**	97.4 (10745/11029)	89.7 (9637/10745)	0.2 (25/10745)	20.7 (2227/10745)
**C6/36, Rep2**	99.7 (10996/11029)	67.5 (7419/10996)	0.3 (29/10996)	8.1 (895/10996)
**Wild-type, In Vitro**	99.5% (10974/11029)	99.0% (10868/10974)	0.3% (30/10974)	36.6% (4016/10974)
**Cyc_Def, In Vitro**	99.0% (10921/11029)	99.5% (10865/10921)	0.3% (28/10921)	45.0% (4916/10921)

Table S3. Pseudoknot coordinates/constraints for structure prediction

**PK_1**		**Region 16 PK**	
**5' Arm, nt Coord.**	**3' Arm, nt Coord.**	**5' Arm, nt Coord.**	**3' Arm, nt Coord.**
753	932	9093	9137
754	931	9094	9136
755	930	9095	9135
756	929	9096	9134
757	928	9097	9133
758	928	9098	9132
759	926	9099	9131
760	925	**DBI PK**	
761	924	**5' Arm, nt Coord.**	**3' Arm, nt Coord.**
**PK_2**		10780	10913
**5' Arm, nt Coord.**	**3' Arm, nt Coord.**	10781	10912
2198	2365	10782	10911
2199	2364	10783	10910
2200	2363	10784	10909
2201	2362	10785	10908
2202	2361	10786	10907
2203	2360	**NS1' PK**	
**PK_3**		**5' Arm, nt Coord.**	**3' Arm, nt Coord.**
**5' Arm, nt Coord.**	**3' Arm, nt Coord.**	3575	3619
3711	4001	3576	3618
3712	4000	3577	3617
3713	3999	3578	3616
3714	3998	3579	3615
3715	3997	3580	3614
3716	3996	3581	3613
**PK_4**		**SLII PK**	
**5' Arm, nt Coord.**	**3' Arm, nt Coord.**	**5' Arm, nt Coord.**	**3' Arm, nt Coord.**
5998	6142	10535	10565
5999	6141	10536	10564
6000	6140	10537	10563
6001	6139	10538	10562
6002	6138	10539	10561
6003	6137	10540	10560
6004	6136	10541	10559
**NS4B'**		**SLIV PK**	
**5' Arm, nt Coord.**	**3' Arm, nt Coord.**	**5' Arm, nt Coord.**	**3' Arm, nt Coord.**
7336	7364	10694	10719
7337	7363	10695	10718
7338	7362	10696	10717
7339	7361	**DBII PK**	
7340	7360	**5' Arm, nt Coord.**	**3' Arm, nt Coord.**
7341	7359	10857	10925
		10858	10924
		10859	10923
		10860	10922
		10861	10921

Table S4. Viral genome sequences used for MSA construction

Virus	Abbreviation	Accession Number
Banzi Virus	BANV	DQ859056
Uganda S Virus	UGSV	DQ859065
Jugra Virus	JUGV	DQ859066
Potiskum Virus	POTV	DQ859067
Saboya Virus	SABV	DQ859062
Bouboui Virus	BOUV	DQ859057
Sepik Virus	SEPV	DQ859063
Wesselsbron Virus	WESSV	DQ859058
Yellow Fever Virus	YFV	JX898878
Ilomantsi Virus	ILOV	KC692067
Donggang Virus	DNGV	NC_016997
Chaoyang Virus	CHOV	FJ883471
Lammi Virus	LAMV	KC692068
Koutango Virus	KOUV	EU082200
Kunjin Virus	KUNV	AY274504
West Nile Virus	WNV	DQ318020.1
Yaounde Virus	YAOV	EU082199
West Nile Virus	WNV	NY99_DB
Alfuy Virus	ALFV	AY898809
Murray Valley Encephalitis Virus	MVEV	NC_000943
Usutu Virus	USUV	NC_006551
Japenese Encephalitis Virus	JEV	NC_001437
Cacipacore Virus	CPCV	KF917536.1
St. Louis Encephalitis Virus	SLEV	NC_007580
Ilheus Virus	ILHV	AY632539
Rocio Virus	ROCV	AY632542
Bagaza Virus	BAGV	AY632545
Israel Turkey Meningoencephalomyelitis Virus	ITV	KC734550.1
Ntaya Virus	NTAV	JX236040
Baiyangdian Virus	BYD	JF312912
Sitiawan Virus	STWV	JC477686
Tembusu Virus	TMUV	JX577685
Naranjal Virus	NJLV	KF917538
Bussuquara Virus	BSQV	NC_009026
Iguape Virus	IGUV	AY632538
Aroa Virus	AROAV	KF917535
Spondweni Virus	SPOV	DQ859064
Zika Virus	ZKV	EU545988
Kedougou Virus	KEDV	DQ859061
Kokobera Virus	KOKV	NC_009029
Stratford Virus	STRV	KM225263
Dengue Virus	DENV_1	EU081265
Dengue Virus	DENV_3	AY648961
Dengue Virus	DENV_2	EU687249
Dengue Virus	DENV_4	AY618991
Nounane Virus	NOUV	EU159426
New Mapoon Virus	NMV	KC788512

Table S5. LNAs used in this study (LNA bases are indicated with a "+")

Region	LNA	Length	LNA Content	%GC	RNA Tm
3'CYC	+T+C+TA+TCC+CAGG+TGT+CAA+T+A+T	20	50.0	40.0	90.0
Region 12	+A+A+TGA+GG+TGT+TGA+TG+TA+A+T+C	20	55	35	87
Region 8	+C+G+TTC+TTA+CAT+TT+TG+GG+T+A+C	20	55	40	88
Region 11	+A+T+TAG+CAC+AA+TCA+TC+AA+G+A+G	20	55	35	83
Region 6	+C+T+TGGC+TG+TC+CAC+CTCT+T+G+C	20	50	60	90
Region 10	+A+T+TCC+CCA+TCCA+TGG+TA+T+A+T	20	50	40	89
Region 16	+G+G+CTC+TGC+TTC+CCT+TGG+C+C+T	20	50	65	94
Non-targeting	+G+T+TTA+TC+TAG+TAA+TAG+ATT+A+C+C	22	50.0	27.3	90
Targeting, highSS	+T+G+ATT+CTG+CTCT+TCA+AA+C+A+T	20	50.0	35.0	88
